# The Hitchhiker’s guide to longitudinal models: A primer on model selection for repeated-measures methods

**DOI:** 10.1016/j.dcn.2023.101281

**Published:** 2023-07-26

**Authors:** Ethan M. McCormick, Michelle L. Byrne, John C. Flournoy, Kathryn L. Mills, Jennifer H. Pfeifer

**Affiliations:** a Methodology & Statistics Department, Institute of Psychology, Leiden University, Leiden, Netherlands; b Department of Psychology and Neuroscience, University of North Carolina, Chapel Hill, United States; c Cognitive Neuroscience Department, Donders Institute for Brain, Cognition and Behavior, Radboud University Medical Center, Nijmegen, Netherlands; d Turner Institute for Brain and Mental Health, School of Psychological Sciences, Monash University, Clayton, Australia; e Department of Psychology, University of Oregon, Eugene, United States; f Department of Psychology, Harvard University, Cambridge, United States

**Keywords:** Longitudinal models, Mixed-effects models, Structural equation models, Nonlinear trajectories, Covariates, Distal outcomes

## Abstract

Longitudinal data are becoming increasingly available in developmental neuroimaging. To maximize the promise of this wealth of information on how biology, behavior, and cognition change over time, there is a need to incorporate broad and rigorous training in longitudinal methods into the repertoire of developmental neuroscientists. Fortunately, these models have an incredibly rich tradition in the broader developmental sciences that we can draw from. Here, we provide a primer on longitudinal models, written in a beginner-friendly (and slightly irreverent) manner, with a particular focus on selecting among different modeling frameworks (e.g., multilevel versus latent curve models) to build the theoretical model of development a researcher wishes to test. Our aims are three-fold: (1) lay out a heuristic framework for longitudinal model selection, (2) build a repository of references that ground each model in its tradition of methodological development and practical implementation with a focus on connecting researchers to resources outside traditional neuroimaging journals, and (3) provide practical resources in the form of a codebook companion demonstrating how to fit these models. These resources together aim to enhance training for the next generation of developmental neuroscientists by providing a solid foundation for future forays into advanced modeling applications.

## 1 Introduction

A variety of longitudinal methods exist to model the course, cause, and consequences of repeated measures across time (Curran et al., 2010). With the advent of large-scale longitudinal data in the field of cognitive neuroscience, researchers are faced with choices as to which method most closely reflects the theoretical model they wish to apply to their data. While individual fields often have methodological preferences, these are often rooted more in tradition than a careful comparison of the available options. Here, we survey a cross-section of longitudinal modeling traditions, starting with a conceptual introduction to each method before considering broad theoretical considerations that motivate model selection for testing a particular theoretically-derived research hypothesis. Through this primer on longitudinal methods, we aim to equip researchers and trainees with a principled approach for adjudicating between available models to best address substantive theory. In other words, to help answer the question “I have longitudinal data, now what do I do with it?” or alternatively, “I plan to collect longitudinal data, which method should I propose in my funding/planning proposal?” We also provide a central reference hub for original empirical and methodological work to guide further reading and training in the specifics of each methodology. In this first section, we outline the aims and structure of this methodological primer and give a general overview of longitudinal methods, before moving into specific models. Two friendly reminders before we begin: (1) **DON’T PANIC**, and (2) know where your towel is.

### 1.1 Aims and scope

In setting forth the scope of this primer, we first need to define clear aims; both for what we hope to accomplish, and topics we will set aside for future discussion. The potential topics related to longitudinal data analysis can (and do) span entire courses, special issues, and books (Hedeker and Gibbons, 2006, Singer and Willett, 2003, Bollen and Curran, 2006, Little, 2013, Grimm et al., 2016), necessitating some limiting principles. We detail these aims and limits below.

*Aim 1*: To provide a decision-tree of criteria for selecting a given method over alternatives when modeling longitudinal data. Many readers will likely have heard of many (if not all) of the models detailed in this primer, however, in-depth training in quantitative methodology is often not available across multiple modeling frameworks for individual researchers and trainees. As such, we provide specific contrasts of the relative strengths, weaknesses, and potential equivalencies both within and between methodologies, focusing on common decision-points in substantive research. These considerations span all facets of the research process, including study design, model parameterization, and inferential support. As such, we seek to not only inform data analysis choices, but the deliberative planning of future studies.

*Aim 2*: To provide reference to a wide variety of primary-source empirical and methodological work from neuro-, behavioral, and quantitative science. While the field of neuroscience has become an increasingly interdisciplinary science (Pfeifer et al., 2018), there remains divides between cognitive neuroscience/neuroimaging and established literatures in the fields of education, development, and applied statistics where longitudinal methods originate. As such, we seek to both highlight exemplary applications of longitudinal methods using neuroscientific data and provide references to methodological papers which provide further detail on specific methods and more-advanced applications that may be of interest. A guiding principle here is accessibility, providing an opportunity for the reader to become an informed user of these methods without being overwhelmed by technical information.

*Aim 3*: To provide a resource of open-access data and code (implemented primarily in R) for testing and training in longitudinal methods. One key barrier to implementing the most appropriate longitudinal method for a given substantive question is often understanding the specifics of model parameterization, output organization, and interpretation. While software comparisons are not the focus of this primer (and often something we will explicitly avoid), some details of popular software options may be relevant to the selection of a modeling approach. While theoretical discussion will largely guide the text of the manuscript, worked examples and the associated code will be provided in an online companion to this primer and referenced where relevant for readers interested in the practical implementation of the models discussed. Files needed to recreate the code companion are available on the Open Science Framework (https://osf.io/bn6yu/).

*Limiting Principles*: We view this primer as an introduction to the decisions that researchers should expect to encounter when modeling longitudinal data. While we attempt to be thorough in our discussion of individual methodologies, we by necessity cannot fully explore the bounds of any one modeling approach. Additionally, while code and worked examples are provided, we similarly cannot replicate formal training courses or specialized tutorials in the scope of a single review. Instead, we provide extensive documentation of primary-source empirical, tutorial, and quantitative work for additional reading (see Aim 2). Some methods we will mostly avoid, either due to their relatively infrequent use in neuroscience applications (e.g., growth mixture models), or due to well-known limitations (e.g., autoregressive panel models, repeated-measures ANOVA) that can be overcome with readily-available modeling approaches. One major exception to this general rationale is the case of intensive longitudinal models. These models have many exciting applications (Bolger and Laurenceau, 2013) but differ in important ways from the longitudinal methods discussed here, and so warrant dedicated treatment of their own.

### 1.2 Longitudinal methods: What are they good for?

**Fig. 1:**
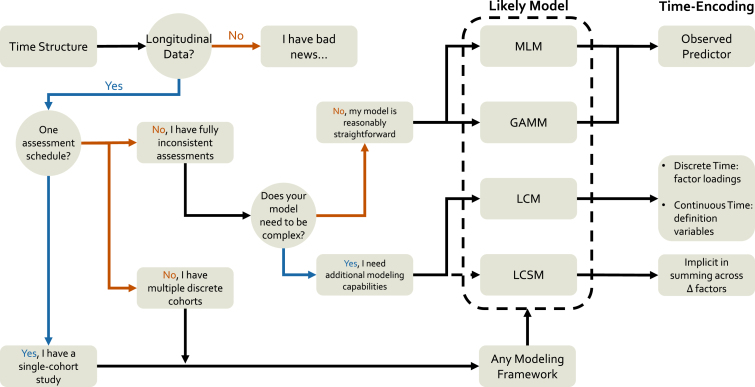
**A decision tree schematic for time-structure model selection considerations**. Model choice for longitudinal data can be complex; however, some rough rules can be mapped out here that serve as an initial guide. Here we focus on decisions about model selection based on assessment schedules and how time is included for each model option. The main feature distinguishing the model frameworks in this section is their ability (or lack thereof) for handling inconsistent assessment schedules. We also highlight how each framework encodes time into the model.

Longitudinal measures, or repeated observations gathered on the same individuals across time, represent a powerful framework for understanding dynamic processes related to the brain and behavior across the lifespan (McArdle, 2009, Sørensen et al., 2021a). Substantive research using longitudinal designs with neuroimaging data span the lifespan, from infant (Cusack et al., 2018, Wen et al., 2019) to aging populations (Kuo et al., 2020, Miller et al., 2016), with a particular focus on the peri-adolescent period (Casey et al., 2018, Mills et al., 2016, Tamnes et al., 2018, Telzer et al., 2018, van Duijvenvoorde et al., 2016). While traditional, annual-observation designs predominate in the literature, longitudinal models are highly flexible and can operate across many timescales, from across months or years to over seconds or minutes (Bolger and Laurenceau, 2013, Hedeker and Gibbons, 2006). Across all of these specifications, however, the focus is on mapping within-unit (usually but not always within-person) change across time (Curran et al., 2014, Curran and Bauer, 2011, Hamaker et al., 2015) as distinct from between-person differences. While oft-repeated, the benefits of longitudinal modeling over cross-sectional approaches to the same theoretical questions are many (Becht and Mills, 2020, Crone and Elzinga, 2015, Curran et al., 2010, Curran and Bauer, 2011, Curran and Willoughby, 2003, King et al., 2018, Kraemer et al., 2000, Louis et al., 1986, Maxwell and Cole, 2007, McCormick, 2021, Molenaar, 2004, Telzer et al., 2018), including increased power to detect effects, the ability to model individual differences in both average level and change over time, and the ability to separate effects to the within- versus between-person level. While we will take these advantages as a given (see Fig. 1, first “No” node), their reality has spurred billions of dollars of investment in the types of data we have come to regard as crucial for understanding how biological, cognitive, social, and behavioral processes unfold across development. Here, we will concern ourselves with theoretical and practical challenges for maximizing the potential of such data, matching our selection of longitudinal models to enable the best testing and refinement of our developmental theories.

### 1.3 Roadmap

The remainder of the primer will take on the following form: First, we will outline the model specifications for four frameworks for longitudinal modeling (Section 2). Once we detail each framework individually, we will then highlight the relative strengths and weakness of each for a number of modeling considerations (Section 3), including how time is included in the model 3.1, how to determine the optimal functional form for the model 3.2, how to include covariates and distal outcomes into models of change 3.3, and how to handle various forms of nested data 3.4. Finally, we touch on how to use the principles discussed here to inform future data collection. And so, without further ado...

## 2 Modeling frameworks

To give us a shared language for discussing various longitudinal models, we first need to introduce each of the four modeling frameworks we will discuss, and outline how they are specified to accommodate longitudinal data. These frameworks fall into two broad categories, **mixed-effects models** and **structural equation models**, which we will take in turn.

### 2.1 Mixed effects models

While there are a number of terms which can be used to refer to the same class of nested data models (including “multilevel”, “hierarchical”, and “mixed-effect”), we will use “mixed-effects models” (MEMs) to refer to the broader group of models that use nested data structures and will encompass more-specific methods. Under this MEM umbrella, we will consider two modeling frameworks, the multilevel (MLM) and generalized additive mixed model (GAMM). Both of these modeling frameworks deal with just-identified models (similar to an OLS regression), meaning that we lack the kinds of absolute model fit tests that we will see in later SEM models. Instead, we need to rely on relative fit indices like the AIC/BIC and likelihood ratio test to assess the fit of a given model. Additional information on model comparisons in MEMs can be found elsewhere (Hamaker et al., 2014, Pu and Niu, 2006, Rights and Sterba, 2020, Stram and Lee, 1994, Vong et al., 2012).

#### 2.1.1 Multilevel models

Multilevel models are the first method for longitudinal analysis that we consider here. Originating in the field of education (Raudenbush and Bryk, 2002), MLMs are some of the most common longitudinal models used in the field of cognitive neuroscience (Braams et al., 2015, Campbell and Feinberg, 2009, Martin et al., 2019, McCormick, 2021, McCormick et al., 2021, Peters et al., 2016, Peters and Crone, 2017, Telzer et al., 2018). Multilevel models were originally developed to deal with the nesting of children within classrooms. Children within classrooms are likely to be systematically more similar to one another than children across classrooms (or schools) because of a wide variety of potential shared characteristics or environments (e.g., school demographics, teacher competency, etc.). This means that children within a classroom do not contribute entirely unique information since they are not a truly random sample and child outcomes like school achievement will be correlated within classrooms (i.e., some classrooms perform higher than others). However, the same insight applies to repeated measurements of the same individual over time (Raudenbush and Bryk, 2002). Some individuals are going to be systematically higher or lower on an outcome (e.g., depression, dmPFC activation) over time and that induces correlations among each individual’s responses. Here we discuss how the MLM is applied to longitudinal data in cognitive neuroscience, and the modeling decisions faced by the researcher. We begin by defining model notation and other key terms, introduce the conceptual framework of longitudinal data analysis in MLMs, and then move into specific features that would inform model choice.

##### 2.1.1.1 Model specification

*Model Equations*: As the name implies, the multilevel model is designed to model data at more than one level, meaning that we have multiple units of measurement that are nested within one another. In longitudinal models, we typically^1^ think of two levels, time (level 1) nested within person (level 2). Variables at level 1 are time-specific observation (i.e., our repeated measures: internalizing, cortical thickness) while variables at level 2 are person-level characteristics that do not vary across time (e.g., biological sex, treatment group). For a simple model with a linear effect of time, we can borrow notation from Curran and Bauer (2011) to express the repeated-measures outcome (  ) for person (i) at time (or occasion; t) as a function of the predictors in the following level 1 equation (note that the colors have no intrinsic meaning; they only provide a visual reference) (see Eq. 1 that is given in Box I).

Where  is the random intercept and  is the random slope for each individual (i). Our predictor  is the observed value of the time-related variable^2^ for each measurement occasion and an individual and time-specific error term (  ) is included to capture the unexplained variance in the outcome. We assume that these residuals are normally-distributed with a mean of zero and a variance of  — in notation form this is  . At level 2 (i.e., the person level), we can write our random intercept and slope as a function of an average (i.e.,  ) and individual (i.e.,  ) effect.^3^ Here we can see this as:

**Figure:**
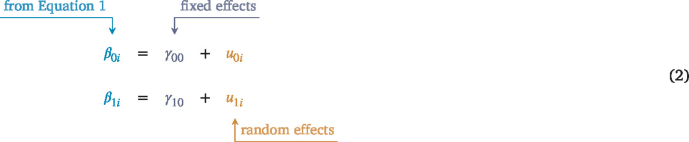

Where  and  are the fixed (or average) effect pooling across individuals and the  and  terms capture the individual-specific (i.e., random) deviations^4^ from that fixed effect. These random effect terms imply that individuals can have higher or lower overall levels of the outcome where time is coded as 0 (i.e., the random intercept,  ; often at the initial time point) and that individuals can show different magnitudes of change over time in the outcome (i.e., the random slope,  ). These level 2 equations can be substituted into level 1 (which is how the model is actually implemented; level 1 and 2 are a conceptual tool) to give us:

**Figure:**
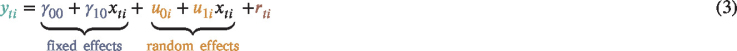

Box I

**Figure:**
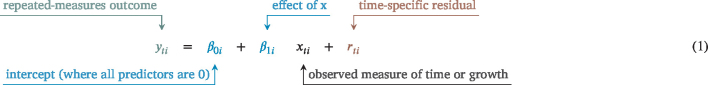

Where the fixed (  ’s) terms represent the average intercept and slope and the random (  ) terms model individual deviations from the fixed effects. One key assumption of the standard MLM is that the random effects are (multivariate) normally distributed. In a model with multiple random effects, we denote this by  ) where  is the vector of random effects and  is the covariance matrix of the random effects. We can express this in matrix form below (note that we only fill in elements on the lower triangle for clarity, but the  matrix is symmetric) (see Eq. 2 that is given in Box II).

In addition to the variances of the random intercept (  ) and random slope (  ), we can estimate the covariance between the random effects (  ). This covariance captures dependence between the intercept (often starting point) and the slope (rate of change over time) across individuals. For instance, perhaps individuals who show lower initial levels show greater increases over time.

Box II

**Figure:**
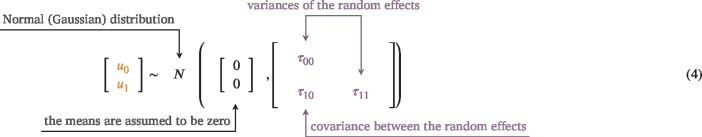

One important thing to point out here is that individual scores for the random effect are not estimated as part of the model, only the variances and covariances of the distributions are parameters; the individual deviations from the fixed effects must be computed on the back-end using model-implied information (we will discuss this later). When our sample size is sufficient^5^ (McNeish, 2017, McNeish and Stapleton, 2016) and the level 2 unit is the individual, this normality assumption is reasonable. However, some units of nesting, most notably individual sites in multi-site studies like ABCD, likely do not meet the theoretical assumptions for a random effect (McNeish et al., 2017) and instead should be modeled using fixed effects approaches (McNeish and Kelley, 2019) where dummy codes for each site are included as separate predictors in the model (for a discussion of the relative trade-offs, see Feaster et al. (2011).

*Residual Structure*: One quirk of the multilevel model is that by default, residuals are assumed to be homoscedastic. In other words, the model obtains a single estimate for the residual variance across all time points. This assumption can be relaxed and heteroscedastic residuals (i.e., different estimates for each time point) can then be obtained. Most major software implementations of MLMs can accommodate heteroscedastic residuals, with the notable exception of *lme4* in R (*nlme* can be used instead).

##### 2.1.1.2 Further reading

Many variations and additional considerations for model specification and estimation exist in the MLM, but this overview orients us to the basics and allows us to move on to additional model features. For those interested in more in-depth explications of the model, details can be found here (Curran and Bauer, 2011, McNeish et al., 2017, Raudenbush and Bryk, 2002, Singer and Willett, 2003). Code for fitting initial MLMs can be found in the Canonical Models chapter of the codebook.

#### 2.1.2 Generalized additive mixed models

##### 2.1.2.1 Model specification

Generalized additive mixed models (GAMMs) share a basic model expression with MLMs. However, rather than modeling the linear effect of predictors (like time), GAMMs allow for the modeling of complex non-linearities in trends over time through the summation of smooth functions. We can see this in equation form in Box III.

Note that instead of a single  estimate for the effect of  on  , there is a generalized function,  , describing the effect (Hastie and Tibshirani, 1987, Lin and Zhang, 1999). We have a lot of flexibility in how we compute this overall function but the general idea is that we generate a set of known functions (e.g., cubic or b-spline functions; Eilers and Marx, 1996, Wood, 2003) across the range of the predictor and then compute estimates of the effect of each function on the outcome across a given set of values within the full range, separated by knot points (for an excellent visual representation of this process, see here). The upshot of this approach is that we can estimate a very complex overall trajectory that has no known mathematical expression as the sum of a set of known functions. In the longitudinal context, this means we can estimate trajectories in outcomes that show complex transitions between increases, decreases, and plateaus across time (Sørensen et al., 2021a, Sørensen et al., 2021b). However, you might have noticed that we are missing  (the random effect of  ) in the equation above. While including a random slope of time is not impossible in theory, it is often not possible in practice for longitudinal studies where the number of observations per person is reasonably small. Compared to other methods we will discuss, GAMMs need a larger range of  values (most commonly age) to estimate the splines over. While in high-density data (e.g., intensive longitudinal data, or some rare traditional longitudinal studies with many time points; Lambert et al., 2001, Sullivan et al., 2015), this can be accomplished within-person, it is likely to be more common in developmental cognitive neuroscience settings to see GAMMs applied in accelerated longitudinal contexts where any individual is only sampled across a small range of possible age values, but different individuals are sample over different sections of the overall age range. This makes GAMMs ideal for lifespan data, where a study might cover multiple decades of life but any one individual is only assessed two or three times (Sørensen et al., 2021a). We will discuss this further in our discussion regarding determining the shapes of trajectories.

Box III

**Figure:**
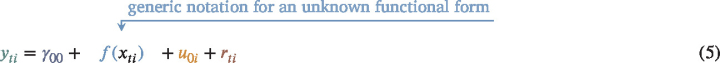

One final point regarding model specification to address is that while GAMMs are characterized by these predictor functions, we are not obliged to use a smooth function for every predictor. We can include a mix of smooth and linear predictors in the same model without issue. Conversely, we can include smooths of compound predictors like interactions where different levels of a moderator variable lead to different smooths on our x variable (see supplemental material in McCormick et al. (2021) for an example in the longitudinal context). We will return to these points in our discussion of predictors and outcomes later.

##### 2.1.2.2 Knot points, “wiggliness”, and overfitting

One key concern with GAMM spline functions is the degree of flexibility we allow in the functional form. Flexibility can be introduced in several ways, including increasing the number of knot points which increases the number of splines being fit, the choice of spline (e.g., cubic versus b-spline), and the degree of “wiggliness” allowed. The first perhaps is the most obvious — increasing the number of non-linear functions fit to the data by including additional knot points will naturally improve the GAMM’s ability to reproduce the average trajectory in the data by fitting unique functions to increasingly local features. The choice of splines is a more complex consideration (for a more in-depth treatment of spline options, see Perperoglou et al. (2019)), but in general, higher-order splines (e.g., b-splines) will increase the flexibility of the GAMM trajectory compared to polynomial splines (e.g., linear or cubic). Finally, the “wiggliness”^6^ of the function is a squared measure of the second derivative — or how much change occurs in the slope of the tangent line (i.e., the first derivative) across different values of the predictor. Functions with more wiggliness will have a greater ability to fit to the data, whereas low wiggliness will smooth over local features in the data (a true line being the least wiggly function). The wiggliness of the function is optimized with penalization as a part of the model fitting to try to reduce function complexity (Wood, 2004, Wood, 2011).

It is easy to see that GAMMs have a high degree of flexibility in fitting the functional form of our data; however, we do need to be concerned that this flexibility will lead to a higher propensity for our model to overfit the sample data. GAMMs straddle the (fuzzy) line between explanatory models like the others we consider here and predictive machine learning models. As such, approaches like cross-validation and sensitivity analysis are especially important with GAMMs to enhance their external validity and generalizability to the population. We will discuss these tradeoffs in greater detail when considering how to establish trajectory shapes.

##### 2.1.2.3 Further reading

Generalized additive models are relatively new for the neurobiological and behavioral sciences, but they have a wide range of uses that may be of interest. Further information can be found here for those interested in practical (Sørensen et al., 2021a, Sullivan et al., 2015) and advanced (Berhane and Tibshirani, 1998, Bringmann et al., 2017, Sørensen et al., 2021b) applications (see the Canonical Models chapter for code examples).

### 2.2 Structural equation models

Like with MEMs, we will use the term “structural equation model” (SEM) to refer to a broad class of models, including latent curve (LCM) and latent change score (LCSM) models. Of course, SEMs encompass still other forms of models (both longitudinal and otherwise), including autoregressive cross-lag panel models (ARCLs), path models, and growth mixture models (GMMs), among others. We will focus on the LCM and LCSM classes of models for this primer rather than these other models, due to a general lack of use in cognitive neuroscience settings (path models and GMMs) or because of known methodological or inferential limitations (e.g., ARCLs; see Curran et al., 2014, Hamaker et al., 2015). Developmental cognitive neuroscience has generally adopted SEM to a lesser extent than MEMs, with the exception of studies in aging. However, SEM approaches are used extensively for modeling longitudinal data in the broader social and behavioral sciences (Bollen and Curran, 2006, King et al., 2018, McArdle, 2009) and there is increasing interest in applying SEM tools to questions in neuroscience contexts.

While we have mentioned previously that MEMs (and especially MLMs) are specialized latent variable models and could fall under the general SEM umbrella (Bauer, 2003, Curran, 2003), there are conventions that tend to differ between SEMs and MEMs due to their historical prevalence/development in different fields. In general, SEMs focus not only on relative fit (e.g., likelihood ratios, AIC/BIC comparisons) but also on measures of “eristics (means and covariances) of the observed (unstructured) data (Bollen et al., 2014, Bollen and Stine, 1992, Hu and Bentler, 1998, Jackson et al., 2009, McNeish and Wolf, 2021, Satorra and Bentler, 2001, Widaman and Thompson, 2003). Furthermore, SEMs are inherently a multivariate modeling framework (even when modeling a single construct; more on this later) and naturally extend to multiple outcomes. In contrast, MEMs are capable of modeling multivariate outcomes (Curran et al., 2023) but only through a data-management/modeling trick that can be challenging to implement appropriately.

#### 2.2.1 Latent curve models

Latent curve models are a class of SEMs that have their origin in confirmatory factor analysis (CFA; Meredith and Tisak, 1990). In CFA, indicators of a construct are used to estimate an underlying latent factor through their shared variance, while isolating item^7^-specific variance that is not shared to the individual item error variance (for an overview of CFA methodology, see Bollen (1989), pp. 227–318). However, an incredibly keen insight came when Meredith and Tisak (1990) used the CFA framework in a highly constrained fashion to allow for estimation of an underlying trajectory in longitudinal data. Now instead of shared variance among a set of items, the latent variables represent features of the longitudinal trajectory (e.g., intercept, linear slope, etc.). We will show how this is achieved below.

##### 2.2.1.1 Model specification

In a confirmatory factor model, we can model multiple (p) items ($y_{pi}$) as a function of the item intercepts ($\nu_{p}$) and the weighted (Λ; the factor loading matrix) contribution of the latent construct ($\eta_{i}$). We can see this below (note we do not include color in this equation because it is not a growth model...yet):

**Figure:**
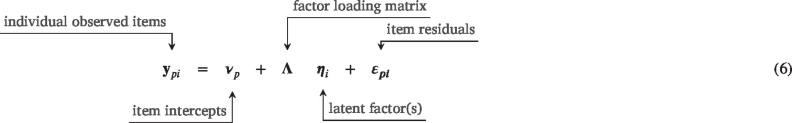

Here the elements of Λ (Lambda; i.e., the effect of the latent construct on the individual item) are freely-estimated parameters (Bollen, 1989). We would represent a factor loading matrix for four items as follows:

(1) $$\Lambda =\left[\begin{array}{c} \lambda_{1}\\ \lambda_{2}\\ \lambda_{3}\\ \lambda_{4} \end{array}\right]$$

In the latent curve model, by contrast, we can model repeated measures of the same item or construct (  ) using a modified form of Eq. (6) (again the colors are just a visual guide, but note that we try to display analogous parameters to the MEMs in the same colors as before) (see equation given in Box IV).

In the LCM, we do not estimate intercepts (ν) of the repeated measures, but instead attempt to reproduce the mean structure – and changes in means over time – through the latent factor and factor loadings (which we will discuss in greater detail below). Furthermore, instead of freely estimating the elements of the factor loading matrix (e.g.,  ), we fix them to particular values to determine the identity of the latent factor (Meredith and Tisak, 1990). So, for a linear slope model, our factor loading matrix would have the form:

(2) $$\Lambda =\left[\begin{array}{cc} 1 & 0\\ 1 & 1\\ 1 & 2\\ 1 & 3 \end{array}\right]$$

The first column of 1’s specifies the first factor (  ) as an intercept while the linearly increasing integers in the second column specifies the second factor (  ) as the linear effect of time on the outcome. Where we choose to place the zero in the second column will impact the estimate of the intercept (more on this in our discussion of centering), but it is typical to place zero at the initial time point to estimate the intercept as the starting level in the growth model (Bollen and Curran, 2006). Each latent factor (  ) can be characterized by two parameters, the factor intercept (  )^8^ and disturbance (  )^9^; we can see this in Box V.

Box IV

**Figure:**
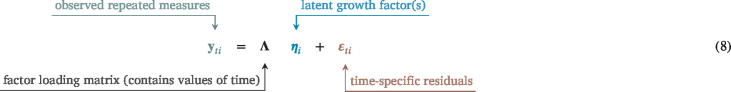

The (co)variance matrix of the disturbances (Psi;  ) allows us to model individual variation around the mean of the factors (  ). For the linear slope model, this is a 4 × 4 matrix:

**Figure:**
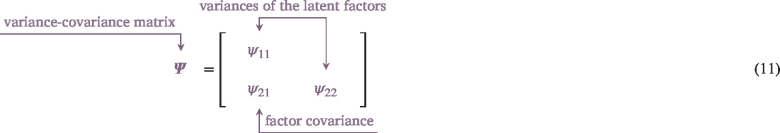

Box V

**Figure:**
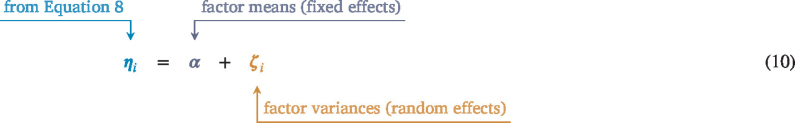

Where  is the variance of the intercept factor,  is the variance of the slope factor, and  is the covariance between the intercept and slope. If all of this seems familiar, this formulation of the LCM gives us the ability to model fixed (i.e., the intercept of the factor,  ) and random (i.e., the variance of the disturbance,  ) effects just like in the MLM (see Eq. (4)). In fact, for a broad class of simple longitudinal models, MLMs and LCMs are numerically identical (Bauer, 2003, Curran, 2003).

One very interesting conceptual insight that SEM can provide is an understanding of how the model reproduces the characteristics of the observed data. The model implied covariance matrix (Σ(θ)) of the repeated measures is modeled as a function of the factor loading matrix (Λ) and factor covariance matrix (Ψ), with the residual matrix (Θ)^10^ accounting for the residual (co)variances (Jöreskog, 1969, Jöreskog, 1970). We can see this in the elegant expression (Bollen, 1989, pp. 85–88, works through the algebraic steps to arrive at these equations in a very clear and accessible fashion):

(3) $$\Sigma \left(\theta \right)=\Lambda \Psi \Lambda^{\prime }+\Theta$$

As mentioned previously, the means of the items are reproduced completely through the factor structure and individual item intercepts are not estimated. The simple expression for the means is as follows:

(4) μ(θ)=Λα

Where the intercepts (i.e., fixed effects) of the latent factors (α) are multiplied by the factor loading matrix (Λ) to give the model-implied means (μ(θ)). When assessing model fit, we compare the estimated model-implied moments (i.e., means and covariances) to the observed moments. Models that fit well will show small discrepancies, while those that fit poorly will do a bad job of reproducing the observed characteristics of the sample data^11^ (Hu and Bentler, 1998, Jackson et al., 2009, McNeish and Wolf, 2021).

*Residual Structure*. In contrast to MEMs, where the default residual structure is homoscedasticity (i.e., a single residual estimate over all time points), the LCM defaults to heteroscedasticity (i.e., a unique residual estimate for each time point). This highlights the truly multivariate nature of the SEM compared with the MEM framework, as each repeated measure is represented as a different variable in the data frame (i.e., the wide format; e.g., Hamaker and Muthén (2020)). Of course, it is trivial to constrain residuals to be equal across time points in the LCM and then compare the two model fits to test (hetero- vs. homoscedasticity) whether the simplification significantly decreases overall model fit using a likelihood ratio test (see the Residual Estimates section of the codebook for how this is done in practice).

##### 2.2.1.2 Further reading

While relatively uncommon in the neuroscience fields, LCMs have been extensively developed and applied in other developmental and aging-related fields. For those interested, theoretical (Biesanz et al., 2004, Bollen and Curran, 2006, Hancock et al., 2001, Hancock and Choi, 2006, Marcoulides, 2018, McArdle, 2009, Meredith and Tisak, 1990, Preacher and Hancock, 2015) and practical applications (Curran et al., 2010, Harden and Tucker-Drob, 2011, King et al., 2018, Moustafa et al., 2021-02-15, Parsons and McCormick, 2022) exist to model many different longitudinal processes that may be of interest (see the Canonical Models chapter of the codebook for examples).

#### 2.2.2 Latent change score models

Latent change scores models are another form of SEMs that, while infrequent in the current developmental cognitive neuroscience literature, have attracted recent attention (Kievit et al., 2018) especially in the context of data with relatively few repeated measures. Interestingly, many longitudinal models (e.g., ARCLs, LCMs) can be reformulated as latent change scores models (for details see Serang et al., 2019). The LCS framework can be expanded quite extensively (Grimm et al., 2012, McArdle, 2009) but we will cover the basic structure here before pointing towards more advanced applications.

##### 2.2.2.1 Model equations

To understand latent change scores, we need to step back and think about where the scores we observe come from (for an excellent review of Classical Test theory, see Bollen (1989), pp. 206–222). Any given observed score (think a behavioral performance metric or a questionnaire response) is composed of “true” score which reflects actual status on that measure and “unique” or “error” variance that can come from a variety of sources (item peculiarities, imprecision, etc.). This can be expressed algebraically as:

(5) $$y_{t,obs}=y_{t,true}+ɛ$$

At the level of true score, the score at any observation can be expressed as a function of a prior time point’s true score and the change in true score between that time point and the current one^12^:

(6) $$y_{t}=y_{t-1}+\Delta y_{t,t-1}$$

Rearranging Eq. (6) allows us then to express the difference in true score as:

(7) $$\Delta y_{t,t-1}=y_{t}-y_{t-1}$$

So if we fix the effect of $y_{t-1}$ on $y_{t}$ (i.e., the autoregressive effect) to a path weight of 1, we can model the residual of $y_{t}$ as a latent difference factor ($\Delta y_{t,t-1}$) that absorbs any changes in true score between observations (for a more thorough walkthrough of these equations, see Ghisletta and McArdle, 2012). When we string together a number of these time-adjacent difference scores, we can then sum (i.e., factor loadings with λ = 1) across the latent difference factors to build a true score trajectory model with an intercept and slope (if this sounds like the LCM, it should). In addition to this overall trajectory model, we can include a proportionality parameter (often denoted β) that allows us to model the latent difference factor as a function of prior status ($\Delta y_{t,t-1}∼\beta *y_{t-1}$). This proportionality effect is one of the more unique features of the latent change score framework (which encompasses many specific versions of the model) and allows for modeling non-linearities in developmental trajectories by inducing exponential trends (Ghisletta and McArdle, 2012, Grimm et al., 2012, Grimm et al., 2013, McArdle, 2009). The inclusion of this proportionality effect is why these models are sometimes referred to as “dual-change” models (i.e., the effect of the overall slope *and* of prior status on latent change). This basic set of equations can be expanded in many interesting ways which are detailed in the advanced topics references (Section 2.2.2.4), but since we focus on the most commonly used version of the model (e.g., Kievit et al., 2018), understanding the basic ideas of the latent and dual change is sufficient for our purposes here.

##### 2.2.2.2 LCSMs and other longitudinal models

As we have mentioned, the LCSM framework can subsume other longitudinal models (see Usami et al. (2019) for a good overview of how many of these longitudinal models interrelate). For instance, significant interest in LCSMs for two-time point data has been generated by the availability of the second wave of ABCD brain data (Henk and Castro-Schilo, 2016, Kievit et al., 2018). LCSMs might seem to be an attractive option in this context (but see Parsons and McCormick, 2022) since we could in theory take advantage of full information maximum likelihood (FIML) to retain cases with missing observations. While this is true to an extent, FIML cannot generate data that does not exist. This means that individuals with only a single observation will contribute to features like the intercept/variance of estimates at those time points but will **not** contribute to the latent difference factor. Indeed, if we were primarily interested in the mean of the latent difference factor (and since this is the effect of time, it is often what is of interest), then that parameter will be identical to a paired-samples t-test. Likewise, the ARCL and LCM can be re-expressed as LCSMs (Grimm et al., 2012) and parameters will be numerically identical.^13^ For the basic versions of these models, the LCSM would be somewhat of an exercise in over-engineering when simpler expressions exist; however, the LCSM expression allows for the inclusion of proportionality effects which cannot be found in the simpler expressions of these models. If the dependence of change on prior status is of interest, then the LCSM is ideal for testing those hypotheses.

##### 2.2.2.3 Measurement error and phantom variables

One peculiarity about the LCSM is that despite the use of latent variable language, LCSMs at their simplest utilize a form of latent variables that differ from more traditional SEM applications. One of the advantages of latent variables is their ability to distinguish between common and unique (or measurement) variance (Bollen, 2002) in a set of p items. In these models, the latent variable is theoretically purged of measurement error and represents a true score that gives rise to the set of items. However, in LCSMs, we can model “latent change” using a single observed variable. In typical applications, this latent variable would be undefined, and so in the LCSM, these single-item factors are often referred to as “phantom” variables, which are essentially a software trick that allows us to model the “residual”^14^ of an item and use it as a predictor or outcome. This trick is accomplished by not estimating an intercept or residual of the item itself and then defining a phantom variable with a loading of 1 so that it copies the parameters of the item up into the phantom. In this context, we cannot really say that the phantom has been purged of measurement error in the same way that we do with multi-item factors. However, if we wish to incorporate this strength of SEMs, we can replace the phantom with a true latent factor, with an associated measurement model (Ferrer et al., 2008), and model latent change on the construct instead of the item level.

##### 2.2.2.4 Further reading

While likely the least familiar to readers from the neurosciences, latent change score models are a broad framework that incorporate and extend many traditional longitudinal applications. Those interested in further details should reference quantitative (Grimm, 2012, Grimm et al., 2012, McArdle, 2009, McArdle et al., 2009, Ram and Grimm, 2007, Usami et al., 2019) and substantive (Ferrer et al., 2007, McArdle and Prindle, 2008, Selig and Preacher, 2009) work using these models (see the Canonical Models chapter for code examples).

## 3 Modeling considerations

Now that we have outlined the four modeling frameworks we will consider here, we can now begin to compare and contrast how they each handle key features of longitudinal data and analysis. We will highlight four broad modeling considerations (with several sub-components): (1) how time is encoded into the model 3.1, (2) how to determine the optimal shape of the developmental trajectory 3.2, (3) how to include covariates (i.e., predictors) and distal outcomes into longitudinal models 3.3, and (4) how nesting is accommodated within each model framework 3.4.

### 3.1 Time structure

A longitudinal model is inherently structured by time (whether or not time is explicitly included in the model) as observations are ordered by their location in the temporal design. However, time structure in longitudinal studies can take many different forms. As is often the case with terminology in the quantitative literature, there is some ambiguity and disagreement about terms. We will attempt to create a logically consistent taxonomy here and elsewhere that we hope can structure the conversation in a useful way. One thing to note is that there may be a distinction between the sampling design used in collecting data and the time coding within a model. We note some discrepancies in these two that might arise in common modeling applications.

#### 3.1.1 Consistent and inconsistent assessment schedules

Before we can run any longitudinal model, we must first collect longitudinal data. How we go about this data collection will constrain many of the downstream modeling options, and researchers should carefully consider the relevant alternatives with reference to their theoretical question. It is far easier to address these issues at the front-end, rather than working around them in the analysis stage. Here we will largely discuss sampling designs with respect to the age of the participants under study. This approach is almost universal in longitudinal designs, however it is important to highlight that these principles could apply to any metric of time — and indeed creative applications are an area ripe for intellectual development in longitudinal modeling.

The most basic design is a cohort study where individuals are assessed repeatedly on the exact^15^ same schedule (see here for a visualization of this kind of design). A classic example would be to assess a class of children across grades; each child is assessed at 6th, 7th, 8th, and 9th grade.^16^ This is the most consistent type of assessment schedule; however, it is often more a function of the modeling approach than a true reflection of a sampling design (since observing everyone at the exact same time is often unrealistic). Here we could code time as t=0,1,2,3 and that would reflect organizing our repeated measures by grade. Of course, individuals might vary in their exact age within a given age category, which we will return to presently. True cohort models benefit from relatively high power due to the pooling of the full sample’s information at each time point and have been used extensively in prior research (e.g., National Longitudinal Survey of Youth, Longitudinal Survey of Australian Youth, Adolescent Brain and Cognitive Development Study). However, these features also impose some limitations for a cohort model, including often being more restricted in overall temporal range (due to practical challenges for observing a full sample across many occasions), confounding of developmental and retest effects (Ferrer et al., 2004, McCormick, 2021), and the assumption that any deviations from the consistent assessment schedule (e.g., age heterogeneity in a study organized by grade) are uninformative noise.

A less consistent version of the cohort design is the cohort-sequential (or multi-cohort) approach (visualized here). In these designs, researchers implement a discrete set of assessment schedules for different cohorts of subjects. To return to the above example, perhaps half of the sample is assessed annually from 6th–8th grade while the other half is assessed from 7th–9th. The advantage here is obvious; we can expand the grade range of the study without observing any more individuals or extending the duration of the study. Of course, this is just one example of such a design and there is a great degree of flexibility in the degree of overlap between the different assessment schedules (see Anderson, 1993, Curran and Bauer, 2011, Duncan et al., 2006, Yang et al., 2021 for some examples; see Curran et al., 2008, Curran and Hussong, 2009 for pooling data across longitudinal studies in this way), but the common feature is that no one individual need be observed across the entire grade range to make inferences across a longer span of time. The time points for a given individual not observed are an example of planned missingness (Little et al., 2014) and can be modeled within a maximum likelihood or Bayesian estimation framework to make use of all available observations and yield unbiased^17^ estimates (Jia et al., 2014, Little and Rhemtulla, 2013, Rhemtulla and Hancock, 2016). For a cohort-sequential design, we still model discrete time points (e.g., grade 6, 7, etc.), which improves the power of estimates for those time points compared with truly inconsistent assessment schedules. However, because not every individual shares the same assessment schedule, we can potentially test for non-developmental effects (e.g., cohort or retest effects) depending on the exact nature of the sampling design (Costa and McCrae, 1982, Ferrer et al., 2004, McCormick, 2021, Sørensen et al., 2021a). This schedule occupies a nice middle ground between the strict cohort design and the (potentially) completely inconsistent accelerated longitudinal design which we will turn to next.

The accelerated longitudinal design is one in which no two individuals need to share the same assessment schedule (see here for an example). The most common form of this design is when we model repeated measures as a function of individuals’ precise chronological age (Braams et al., 2015, McCormick et al., 2021, Mehta and Neale, 2005, Mills et al., 2016, Peters and Crone, 2017, Sørensen et al., 2021a, Zhou et al., 2015). In our example, we could model individual responses as a function of age instead of grade, which would actually give a uniform distribution of assessment timing within grade (since the oldest in one grade would be only days younger than the youngest in the next grade). However, in this example, the age range is not extended, merely the density of time points is increased due to the individually-varying assessment schedules (some individuals are assessed at t=12.1,13.1,14.1, while others are assessed at t=12.67,13.67,14.67, etc.). However, a common application of the accelerated longitudinal design is to expand the age range under consideration to an even greater extent than is possible with the cohort-sequential design. For instance, we might be able to sample from ages 8–29 over a 5-year study period (Braams et al., 2015, McCormick et al., 2021, Peters and Crone, 2017) using such a design. The flexibility of the accelerated approach is naturally attractive; however, this design introduces the greatest divergence of the longitudinal models we might consider fitting as the manner in which the different models incorporate time becomes relevant. However, one additional limitation of this sort of assessment schedule design is that the estimate of the effect at any given age is markedly reduced (and indeed not directly estimated) because we cannot pool information across individuals. Additionally, accelerated longitudinal studies almost always have lower sample density towards the tails of the age distribution, making model results potentially sensitive to small number of observations at these tails.

#### 3.1.2 Time coding

Before we explore the approaches that each model takes for including time information into the modeling of brain and behaviors, we first need to explicate how we will code time to support our inferences. Of primary concern is where the intercept is estimated, but other considerations are addressed. We will consider time coding in the context of a linear slope model before generalizing these principles to higher-order polynomial models.

Just like in any linear model, the model intercept is defined as the value of the outcome where all other predictors are zero (Bollen and Curran, 2006). If we wish to meaningfully interpret the intercept, we need to ensure that the scale location where the other predictors are zero is also meaningful. This is most often accomplished by centering or normalizing predictors to a central tendency (mean or median) or minimum value so that the intercept is at the mean or minimum of the other predictors, although other approaches may be appropriate (Aiken and West, 1991, King et al., 2018, McCormick et al., 2021). In a longitudinal model, one of these other predictors is time and where we code time as zero becomes the estimated value for the intercept. The overwhelmingly common practice is to place zero at the first time point (e.g., t=[0,1,2,…]) such that the estimated value is the “starting point” for the outcome of interest. However, there is enormous flexibility with the coding of time (Biesanz et al., 2004, Grimm, 2012, McCormick et al., 2021, Mills et al., 2014). If we want to estimate intercept variability at the end of a treatment study, we could place the zero-point at the final time point (e.g., t=[…,−2,−1,0]). With each coding scheme, we get different estimates for the intercept^18^ since it reflects the fixed and random effects of the level of the outcome of interest at different points in the overall trajectory,^19^ and the effects of predictors on the intercept will alter accordingly with this change (Biesanz et al., 2004; we discuss predictors in Section 3.3.1). While this might appear like we are estimating different models when we change the time coding, in fact, all of these models are **exactly** likelihood-equivalent; we can even transform each solution into one another if we choose (Biesanz et al., 2004). So it is possible to estimate a model with a single time-coding scheme and then generate alternative estimates at any time point using only the information contained in that one solution (Biesanz et al., 2004, Hancock and Choi, 2006). This does not take away from the potential utility of one coding scheme over another for *interpretation*, but it is key that we recognize that changing time coding schemes only draws information from the exact same data and so the fundamental information contained in the model is not unique across different codings. See the Time Structure chapter for examples of this point.

While we have focused on the changing estimates for the intercept depending on where we locate zero, what has been happening with the slope? As may be intuitive, changing the time coding in a linear model will not change the estimate of the linear slope at all. Indeed, this will generalize to higher-order polynomials, where the highest order effect (e.g., quadratic, cubic, etc.) will be unaffected by changes in time coding (Biesanz et al., 2004). However, lower-order effects (e.g., the linear effect in a quadratic model) will show differences in their estimates depending on changes in the time coding. This still does not reflect a change in the underlying model information and the models will be likelihood equivalent, but there are more things to keep track of in these higher-order models.

Finally, one thing to take caution in is that the zero point in longitudinal models should be contained within the range of the data. Of course, this is true of any linear predictor, however, we often place special interpretational weight on the intercept in longitudinal models. For instance, in a study of 6 – 18 year olds, using the raw ages (t=[6,…,18]) will result in an intercept estimate not for 6 year olds, but for 0 year-olds. While the model can produce an estimate for this hypothetical point in the age distribution, we could not make internally or externally valid inferences on this estimate. Instead, we would want to use an alternative time coding to estimate the intercept at a meaningful point within the observed time window; for instance, a coding of t−6 ($t^{*}=\left[0,\ldots ,12\right]$) to estimate the intercept at the earliest age in our sample. Remember that polynomial growth functions hypothetically extend to ±∞, but we should bound our inferences within the range of the data available to us (Hancock and Choi, 2006).

##### 3.1.2.1 Model comparisons

*Mixed-Effects Models*. Multilevel and generalized additive models include time similarly and so we will refer to them generally and point out specific differences as they arise. However, with respect to how the effect of time is expressed in the model, these two approaches are identical. Indeed, nothing much special is happening from the model’s perspective. Time is simply another predictor that enters the model linearly as any other (e.g., stress, task performance) would. As such, although we conceptually distinguish longitudinal models from others in the mixed-effects framework, no special estimation approach is needed compared with models on cross-sectional data. But before we feel too let down, we must recognize that this is the **strength** of the mixed-effects models. Because time is treated like any other predictor, we can accommodate almost any^20^ type of time structure in our data without issue. So fully inconsistent assessment schedules like those in accelerated longitudinal designs present no challenge for mixed-effects models because we do not need individuals to share values of the predictor (if you are confused, think about another predictor like depression and whether you would be concerned that individuals do not share the same values; you would not be). As such, including exact ages for each participant is entirely possible (and should likely be the default approach for estimating developmental effects with age) instead of needing to bin ages into discrete units. This removes error variance due to the compression (or the technical term “smooshing”) of age heterogeneity when estimating the model.

*Latent Curve Model*. In contrast to the mixed-effects model, time does not appear explicitly as a predictor in the model for the LCM or LCSM. Rather, time is coded into the factor loading matrix (Λ) which will weight the contribution of the underlying latent factors (η). The LCM is a highly-restricted form of the confirmatory factor model (CFA) where the factor loadings are set prior to estimation rather than being freely estimated (Meredith and Tisak, 1990). As mentioned before, the insight that time structured data can be modeled in this way is an incredibly important one, allowing longitudinal analysis access to the full flexibility and strength of the structural equation modeling framework. However, in its traditional form, the LCM has some limitations in the kinds of time structures it can accommodate. More recent developments allow us to overcome some of these limitations, but they introduce some trade-offs (although perhaps not as many as is often thought).

The primary limitation of the factor loading approach is that the traditional LCM attempts to model a residual estimate for each discrete repeated measure separately (Bollen and Curran, 2006, Curran et al., 2010). As such, the LCM pools information across individuals in order to compute a unique residual. In this form, we need some consistency (by design and/or through compressing information in the model) in the assessment schedule (e.g., a time 1, time 2, etc.). We are not limited to the fully consistent cohort model, as the full information maximum likelihood estimator used will allow for the cohort-sequential design where the time points where individuals were not assessed by design are treated as missing (Little and Rhemtulla, 2013). As such, a long-standing “truth” was that accelerated longitudinal designs were the sole province of mixed-effect models, because the individually-varying assessment schedule did not allow these unique residual estimates.

While the second point is true, this does not prevent us from estimating a longitudinal model on accelerated data using the LCM framework. Rather than having a single unified factor loading matrix for the entire sample, we can code individual factor loading matrices. Known as definition variables (Mehta and Neale, 2005, Mehta and West, 2000; or TSCORES in Mplus), these methods allow us to accommodate fully inconsistent assessment schedules.^21^ The downside is that this approach prevents the computation of absolute measures of model fit like the CFI/TLI/RMSEA because of the lack of an appropriate baseline model to compare with our model’s fit (Mehta and West, 2000). Of course, this is a limitation we accept every time we fit a mixed-effect model^22^ (Curran, 2003) so perhaps this should not be treated as the end of the world; after all we did choose a complex structure of time with many other advantages to weigh against this loss. Currently, the definition variable approaches are relatively specialized and have yet to be incorporated in all software options (OpenMx [von Oertzen et al., 2015] and Mplus implement these models, but at the time of this writing, *lavaan* has yet to include that functionality; example Mplus syntax files are available here).

*Latent Change Score Model*. Finally, the latent change score is perhaps the most unintuitive in terms of how it structures data in time. Interestingly, the values of time appear nowhere in the LCSM model, either as a predictor or in a factor loading matrix. Instead, the slope factor in a linear LCSM sums (i.e., all factor loadings are 1) across the latent change (Δ) factors built between each time point rather than using an increasing factor weight like is done in the LCM. As such, LCS models are generally limited to cohort or cohort-sequential types of structures, as the individually varying assessments cannot be represented easily^23^ within the model structure (but see Estrada et al. (2022) for recent developments).

#### 3.1.3 Decision tree I

With these model comparisons in mind, we can create a rough decision tree for model selection with respect to time structure. As can be seen in Fig. 1, the primary consideration which guides model selection is the consistency of assessments. On one end, single- or multiple-cohort studies with highly-consistent assessments can be readily modeled with any of the four frameworks, and other considerations (which we cover in subsequent sections) should drive model selection. However, highly inconsistent schedules would suggest leaning toward mixed-effects models unless there was a compelling need for additional modeling options available in the structural equation models.

#### 3.1.4 Additional considerations

While this manuscript is primarily concerned with model comparisons, we also highlight some additional considerations that may aid in modeling longitudinal data.^24^ We will highlight a selection, but this should not be taken as an exhaustive list.

##### 3.1.4.1 Different forms of “time”

In the overwhelming majority of longitudinal models, time is represented by some rough approximation of the amount of time an individual has spent on this Earth. Whether age, grade, or some other chronologically structured metric, these metrics assess the per unit change in the outcome across minutes/hours/years. However, reflecting on the majority of developmental theories, chronologically-based metrics might be the least relevant in many situations. For instance, many theories (Casey, 2015, van Duijvenvoorde et al., 2016, Wierenga et al., 2018) posit change due to biological maturation, a process that roughly tracks chronological time – but certainly not exactly – and often varies widely in timing and tempo across individuals (Marceau et al., 2011). Other theories might suggest that changes in brain and behavior are driven by retest effects (e.g., learning or habituation; Ferrer et al., 2004, McCormick, 2021, McCormick et al., 2021) which may or may not be consistent across individuals. As such, using age to structure longitudinal models will lead to crude and biased inferences about the developmental processes under study (McCormick, 2021).

In developmental neuroscience, perhaps the most obvious alternative to age in a longitudinal model is pubertal development (McCormick, 2021, Wierenga et al., 2018), while in lifespan work, probing for retest effects (either through a model or design) that can partially counteract age-related declines are common (Ferrer et al., 2004). One approach might be to ignore age and simply have pubertal status (or other variable) be the sole form of time (Wierenga et al., 2018), but it is also possible to utilize planned missingness designs to recover unbiased estimates of multiple forms of time simultaneously (e.g., age and puberty; Goddings et al., 2014, McCormick, 2021); see here for examples. Of course, we should not ignore that there are often tradeoffs in utilizing these more theoretically relevant forms of time. Phenomena like maturation are incredibly complicated, with a multitude of components (e.g., hormone production, physical development, neural plasticity) that may be difficult (or impossible) to distill into one or a small set of temporal predictors. Furthermore, these components may have higher levels of measurement error associated with them than the relatively straightforward measure of chronological age. One the other hand, giving up because things are hard is not the solution either. While still relatively nascent in their development, measures like “brain age” may offer a way forward. Measures of “brain age” attempt to predict how old we would expect an individual to be based on some set of features (e.g., morphological and functional features of the brain; Cole and Franke, 2017). While using a metric of “brain age” to model longitudinal changes in the brain might need to address issues of circularity, one could imagine using a similar idea to predict maturational status during puberty or senescence based on non-neural features to subsequently structure a longitudinal model for outcomes of interest. One major challenge for this kind of approach is to identify a gold-standard validated measure of maturation to evaluate the predictive model before application to a new sample.

One final alternative is to create structures of time using information outside of the model. One natural example of this approach would be relevant in studies where transitions occur inconsistently across individuals. Say we are interested in reward system reactivity following the initiation of substance use in adolescence, where there will be natural variation in the chronological age of onset. Instead of centering time to a given age time point for all individuals, we could instead center within a person to the time point that they first report substance use. So, for an example study, we might have some individuals who begin earlier (e.g., t=[−1,0,1,2,3]) or later (e.g., t=[−4,−3,−2,−1,0]). Time here is now scaled in “years until substance use initiation” instead of chronological age. Note that this is not examining different trajectories pre- and post-initiation like in a piecewise linear approach (e.g., Flora (2008)), but rather re-scaling time for each individual separately to center on a meaningful event (e.g., time-to-death in studies of aging; Kurland et al., 2009). While not as common in longitudinal studies compared to universal time coding approaches, this is an application of well-known approaches to centering of other predictors in longitudinal models (Biesanz et al., 2004, Curran and Bauer, 2011).

##### 3.1.4.2 Residual estimates

One modeling note that should be considered when fitting longitudinal models across different methods is the default model behavior when it comes to estimating residual structures.^25^ In mixed effects models (MLMs and GAMMs), the default is to estimate homoscedastic residuals or to generate a single estimate of residual variance pooled across time points. In contrast, the default for structural equation models (LCMs and LCSMs) is to estimate a unique residual variance for each time point (i.e., heteroscedastic residuals). However, these defaults are only that, and the majority of software programs allow for either specification.^26^ It should be noted that homoscedasticity is a model constraint that could introduce bias into the model if improperly imposed. Fortunately, the homoscedastic model is nested within the heteroscedastic model and the decrement in fit associated with the imposition of homoscedasticity can be assessed using a likelihood ratio test (see here for testing these competing models).

### 3.2 The shape of development

In our tripartite goals of development (Curran et al., 2010), the first is to chart the course of development. In other words, we need to establish the optimal shape of the developmental trajectories for the construct under study in our sample. However, there are a myriad of potential shapes of development, and that shape may not be consistent across individuals or discrete groups. Furthermore, different modeling frameworks allow for more or less flexibility in specifying different functional forms to developmental trajectories. In this section, we review the broad classes of potential developmental trajectories that one could fit to their data, beginning with highly constrained polynomial models and working our way up a hierarchy of flexibility towards truly non-linear models. We highlight the relative strengths of each modeling framework along the way, and then end with a discussion of heterogeneity and generalizability across samples.

#### 3.2.1 Polynomials

Leaving aside intercept-only models (Curran et al., 2014) which are more common in intensive longitudinal modeling, the simplest form a developmental trajectory can assume is a line. While simple, linear growth models form the backbone of longitudinal modeling and are often reasonable models for the kinds of data we frequently collect. Furthermore, the linear model is easily fit with all of the modeling frameworks we discuss here.^27^ Of course, linear models are simply the canonical example of the broader family of polynomial models. While less frequent, higher-order models like quadratic (Braams et al., 2015, McCormick et al., 2021, Peters and Crone, 2017, Tamnes et al., 2018), cubic (Chassin et al., 2009, Herting et al., 2018, Mills et al., 2016), or things like inverse models (Luna et al., 2004, Nelder, 1966) also fall under the polynomial umbrella, where developmental trajectories are specified using powered terms of time.^28^ While these likely cover the overwhelming majority of current applications, there is nothing stopping us from adopting even more exotic polynomial models if we think that they may be relevant (and we have the time points to support them; Preacher and Hancock, 2015). In all cases, no matter how complex the functional form a given model implies, the values of time are fixed and known in the model. Consider the following factor loading matrices for higher-order latent curve models (here we will focus on the LCM notation because it is nicely compact, but the same principles logically apply to the other model frameworks).

(8) $$\Lambda_{lin}=\left[\begin{array}{cc} 1 & 0\\ 1 & 1\\ 1 & 2\\ 1 & 3\\ 1 & 4 \end{array}\right]\;\Lambda_{quad}=\left[\begin{array}{ccc} 1 & 0 & 0\\ 1 & 1 & 1\\ 1 & 2 & 4\\ 1 & 3 & 9\\ 1 & 4 & 16 \end{array}\right]\;\Lambda_{cub}=\left[\begin{array}{cccc} 1 & 0 & 0 & 0\\ 1 & 1 & 1 & 1\\ 1 & 2 & 4 & 8\\ 1 & 3 & 9 & 27\\ 1 & 4 & 16 & 64 \end{array}\right]\;\Lambda_{inv}=\left[\begin{array}{ccc} 1 & 0 & 0\\ 1 & 1 & 1/2\\ 1 & 2 & 2/3\\ 1 & 3 & 3/4\\ 1 & 4 & 4/5 \end{array}\right]$$

We can see that for each increase in the polynomial order (linear – cubic), we add an additional predictor with higher-powered terms of the linear model. However, in each case, we know the **exact** values for each time predictor that models the shape of the particular developmental trajectory (i.e., no values are estimated). Indeed, columns three and four in the cubic model are just the squared and cubed values of the second column, and we simply add a 1/x term to the matrix for the inverse model. In SEMs, these factor loading matrices are used to identify the latent variables that are associated with them while in MEMs we would have variables in our data frame with these values for each individual (see code examples in The Shape of Development chapter for more information). The fixed-and-known nature of the time predictors in polynomials lends it both power and restrictions for modeling developmental trajectories. Because of their highly constrained nature, polynomial models are often incredibly easy to fit to a wide variety of data and achieve reasonable measures of model fit. They also offer incredibly natural interpretations of model parameters, because change per unit time is expressed in an easily understandable form. On the other hand, the constraints of polynomial models often limit their ability to describe complex patterns of development, or to account for long periods of change (Fjell et al., 2010, Sørensen et al., 2021a, Tamnes et al., 2017).

It is worth a moment to step back and consider the nature of polynomials to see how they might provide sub-optimal fit for describing developmental processes. First, all polynomials are defined across the range of $\left[-\infty ,\infty \right]$. While the careful researcher would only ever use the function to infer information within the range of the sample data,^29^ this mathematical definition still influences how developmental trajectories are estimated. Consider for example, a quadratic model for data that is truly linear. Simply due to the mathematics of including the higher-order term, slight curvature will be induced. Furthermore, as the developmental window expands, the less well-described outcomes are by simple polynomials. For instance, how likely is it that reward sensitivity continues to show permanent increases across the whole lifespan, even if trajectories of change are fit well by a linear term *during adolescence*? Or that the negative values of emotional regulation that a quadratic form will eventually imply are reasonable? As such, the types of inferences we can make with these models are much more limited in lifespan types of data.

However, with quadratic (and cubic) terms in particular, an even more problematic issue is how the inflection points in developmental curves are dependent on cases at the edges of developmental trajectories. For instance, in data that increases before plateauing, a quadratic function will attempt to fit a model that shows decreases at later ages because that is the shape of a quadratic. Because this form is forced in the polynomial model, observations at the tails of the age range will exert extra influence on the curvature in ways that may be undesirable (Fjell et al., 2010). For this reason, researchers would do well to include robustness checks on higher-order polynomials by running permutations of the model with different subsamples of individuals at the edges and assessing the changes to the effects of interest. While these limitations are unlikely to (and should not) prevent the widespread use of polynomial models for modeling longitudinal change, researchers should be aware of the mathematical assumptions they bring on board when using polynomial expressions. At the end of this section, we discuss some potential ways forward, combining multiple approaches in order to provide greater confidence in results.

#### 3.2.2 Piecewise models

One potential compromise for fitting more complex developmental trajectories (e.g., changes followed by plateaus) without sacrificing interpretability of the parameters is to use piecewise functions (Flora, 2008). Piecewise functions allow us to fit a set of simple polynomial models to portions of the overall developmental trajectory, joined by knots which allow for different kinds of discontinuities in the functions. Returning to our factor loading matrices from before, if we thought that our developmental trajectory was best described by initial increases followed by some plateau, we could fit two linear pieces using the following form.

(9) $$\Lambda_{piecewise}=\left[\begin{array}{ccc} 1 & 0 & 0\\ 1 & 1 & 0\\ 1 & 2 & 0\\ 1 & 2 & 1\\ 1 & 2 & 2 \end{array}\right]\;$$

In this specification, the first piece (second column) is a linear effect over the first three time points and then no further (this is why the integer increases stop). The second piece (third column) has no effect for the first two time points and then begins exerting an influence for the last 3. As you can see, the two effects share the third time point (i.e., the knot point) which is where the discontinuity in the overall functional form occurs. The above specification (known as the two-rate parameterization) allows us to interpret the effect of the two pieces quite intuitively for most contexts (each piece is the per time unit change in the outcome) however, it is possible to formulate the piecewise another way.

(10) $$\Lambda_{piecewise^{\prime }}=\left[\begin{array}{ccc} 1 & 0 & 0\\ 1 & 1 & 0\\ 1 & 2 & 0\\ 1 & 3 & 1\\ 1 & 4 & 2 \end{array}\right]\;$$

Here, in what is known as the added-rate parameterization, we can now interpret the second slope as the per time unit *deflection* from the initial slope (i.e., the additive effect of the first and second rates). This parameterization is relatively rare but can be well-suited for intervention research where we might want to understand how treatment deflects individuals from their original trajectories. Like the standard polynomial model, both of these parameterizations are easily fit using MEMs or SEMs (code examples of the MLM and LCM forms of these models can be seen here; GAMM and LCSM versions are possible but uncommon given their ability to model true non-linearities in other ways). Of course, the linear piecewise model is just the most simple version to consider. Given sufficient numbers of time points, we could model higher order functional forms on each side of the knot and indeed can fit different forms for each piece (e.g., a quadratic first piece followed by a linear second piece; Cudeck and Klebe, 2002, McNeish et al., 2021).

One key feature of the piecewise model is the knot point, where the functions are joined. Since a line is minimally defined by three time points, we need a minimum of five observation occasions to fit the simplest form of these models (3 for each piece with a shared time point at the knot), which may limit their application for practical reasons. While placing the intercept of the model at the initial time point may be perfectly reasonable, researchers often wish to estimate the level at the transition (i.e., knot) point in the trajectory, which involves the simple re-coding of the first time predictor, as we can see below.

(11) $$\Lambda =\left[\begin{array}{ccc} 1 & -2 & 0\\ 1 & -1 & 0\\ 1 & 0 & 0\\ 1 & 0 & 1\\ 1 & 0 & 2 \end{array}\right]\;$$

For instance, we might wish to estimate symptom severity at the start of the intervention or children’s risk preferences at the start of a school transition (e.g., middle to high school), making this coding of time the most informative. In other contexts, however, we might not know exactly when a transition will occur (e.g., when one begins to use a given substance). In these instances, we can add an additional set of parameters that will model the unknown location of the knot point (Cudeck and Klebe, 2002, Kohli et al., 2013). Of course, these methods often require many more time points to arrive at stable solutions, and the locations of knots are fundamentally limited by the number of time points (i.e., the knot can never be placed at the first or last two time points). As such, these models may be more appropriate in designs that either have denser sampling or cover a larger age range using accelerated designs (see McCormick et al. (2021) for an example of combining piecewise models for denser samples with simpler polynomial models in these types of designs).

#### 3.2.3 Nonlinear models

Finally, we can consider models which fit truly nonlinear patterns of development over time.^30^ We will exclude nonlinear trends based on polynomials from this discussion for reasons that will hopefully be clear, but it should be noted that our hierarchy is not entirely without some fuzziness. Up until now, the models we have discussed can mostly be fit with whichever modeling framework the researcher desires. However, here there is much greater need to carefully weigh the different applications that each method may be best suited for. We first discuss the methods each framework takes to model non-linear patterns over time and the specific attendant considerations before moving into a discussion of the overall strengths and challenges of non-linear trajectory approaches.

##### 3.2.3.1 Mems

The majority of the nonlinear applications (again excepting the polynomial models) in MLMs are those which are nonlinear with respect to the parameters (e.g., a logistic or negatively-accelerated exponential model; see Cudeck and Harring, 2007, Grimm and Ram, 2009, Harring and Blozis, 2014 for examples). While certainly interesting in their applications, they do not differ much in principle from linear models with respect to their flexibility of fitting developmental trajectories. Just like standard polynomial models, the researcher needs to pre-specify the functional form and then the various parameters associated with that form are estimated as part of the model fitting-procedure. This stands in strong contrast with GAMM, where there is substantial flexibility in fitting developmental trajectories that cannot be described by a single, unified equation. Indeed in a GAMM, the trajectory is built up from several splines or basis functions which combine to form a highly complex nonlinear surface (Lin and Zhang, 1999, Sørensen et al., 2021a, Wood, 2011). As such, GAMMs are one of the best models for fitting data which contains transitions between periods of change and periods of stability or reversals in the direction of change, which is often true of complex intensive longitudinal data, as well as lifespan data (Sørensen et al., 2021a, Tamnes et al., 2017) where continual growth in any direction is unlikely to be realistic.

##### 3.2.3.2 Sems

Turning to SEMs, there are several interesting potential nonlinear models that are possible. The LCM can accommodate all of the specified nonlinear functions that are possible in the MLM (see Bauer, 2003, Curran, 2003, Preacher and Hancock, 2015 for some bridges between these models), however, the change in parameterization from time as an observed predictor to being an element in the factor loading matrix allows for a unique form of nonlinear model. In what is known as a free-loading or latent-basis model (McArdle, 2009), we can return the LCM to some of its confirmatory factor analytic roots and estimate rather than specify some subset of factor loadings. We can implement this model in one of two ways, shown below.

**Figure:**
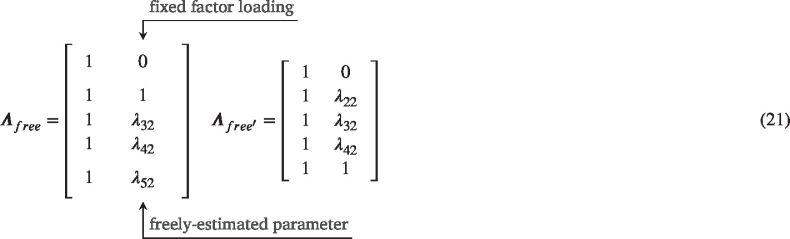

Here we can estimate all but two factor loadings, which set the scale of the growth model, based on the characteristics of the data (see here for implementing these models). While the former parameterization scales the estimated loadings to the amount of change between time 1 and 2, the former assesses how much of the total change between time 1 and 5 has occurred at each time point. In both, however, we have increased the flexibility of the model to accommodate nonlinearities by allowing for *unequal* change between sets of observations (Debatin et al., 2019, McArdle, 2009). This type of model might be especially useful if a researcher expects there to be variability in the rate of development over time. For instance, when examining the developmental trajectory of peer influence, the COVID pandemic might interrupt more systematic growth we might have seen otherwise. While we will talk about general challenges associated with these flexible nonlinear models below, one specific challenge that should be raised here is the challenge that free-loading models present for parameter interpretation. In the usual linear LCM, the fixed and random effects are easily interpreted as the average and individual change respectively in the outcome per 1-unit increment in time. However, in the free-loading model, the unequal change limits us somewhat to talking about the degree to which the fixed effect is expressed in individual effects. While very flexible, this may be a somewhat unsatisfying limitation when interpreting results. The LCS model, by contrast, typically implements nonlinearities into developmental trajectories not through the factor loading matrix (although in theory this is possible, exactly what those parameters would mean in the larger context of the model has not be explored in depth) but through the inclusion of the proportionality parameter (for details, see Section 2.2.2 on the LCSM; Grimm et al., 2012). This parameter can be thought of as a “dampening” – or “exploding” if it accelerates the function – parameter which introduces an exponential form to the trajectory, making the LCSM ideally suited for data with asymptotic growth patterns. Because the LCSM can subsume the LCM, it can be viewed as the most maximally flexible form of the SEM and its applications for modeling nonlinearities is an active area of research (Grimm et al., 2012, Grimm et al., 2013, Grimm and Ram, 2009, Ram and Grimm, 2007).

##### 3.2.3.3 Advantages and challenges

As we have mentioned several times, the true power of these nonlinear models is the ability to flexibly fit complex, non-monotonic changes. These approaches have become very appealing to researchers who feel that we often know relatively little *a priori* about the shape of development and who would prefer a data-driven approach where the characteristics of the data are given more weight in determining developmental trajectories. To some extent, this is a perfectly legitimate approach, as many of these models do a good job of approximating the local features of sample data. However, the idea that these data-driven approaches can replace more theoretically informed forms of trajectories is likely ill-conceived both practically and theoretically. Given the complexity of the trajectories that these models fit and the relative lack of interpretable individual effects, they are most often not useful as explanatory models and instead are most useful as descriptive or purely predictive^31^ models. Furthermore, these models have a terrible tendency to overfit the local features of the data and can appear to be the best-fitting model even in simulations where the true data-generating mechanism is known to be otherwise, simply due to optimizing to sampling variability. As such, we would encourage researchers who adopt these methods to accompany them with sensitivity analyses such as out-of-sample replication or a form of cross-validation (e.g., split-half or k-fold; Grimm et al., 2017, Jacobucci et al., 2021, de Rooij and Weeda, 2020) to ensure they are not overfitting the data at hand.

#### 3.2.4 Decision tree II

**Fig. 2:**
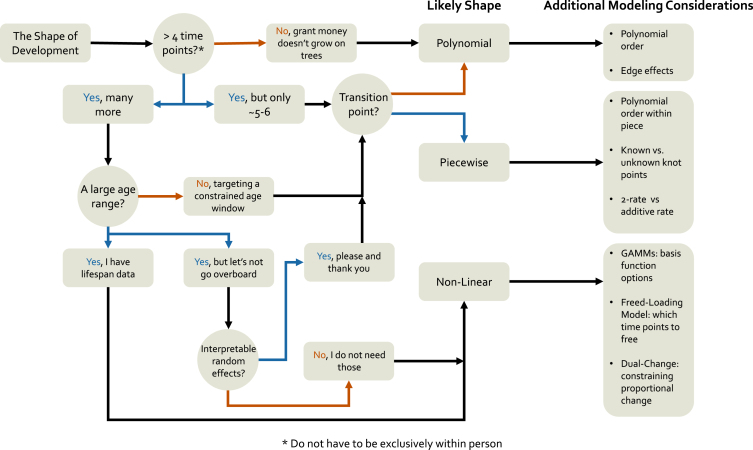
**A decision tree schematic for determining the shape of development**. The complexity of developmental trajectories we can model is determined by both the number of observations and the range of development we attempt to model. This rough heuristic can give some ideas of where to begin with establishing the optimal developmental shape. Note that the number of time points does not need to be exclusively within-person (e.g., multi-cohort or accelerated data).

Before moving into some additional considerations for determining the shapes of trajectories, we can summarize a decision tree for adopting different trajectory shapes for our data. The major decision points hinge on both the number of observations (either within or across person), the developmental window covered by the data at hand, and the need for interpretable parameters (see Fig. 2 for a flow-chart of these considerations). While many traditional longitudinal designs can be well-described by simple polynomial models, with more time points and greater developmental coverage (e.g., a larger age range), nonlinear models become more attractive. However, we need to be concerned about overfitting and recovering parameters with straightforward developmental interpretations when adopting these models.

#### 3.2.5 Additional considerations

Here we briefly outline some additional considerations that researchers should keep in mind when establishing optimal functional forms in their developmental trajectories. Some of this information builds on some briefly-mentioned points from above, but with an eye towards comparing across approaches.

##### 3.2.5.1 Fixed versus random effects

In most of the models we have discussed thus far, we can model two types of effects in our model, the average or typical (i.e., fixed) effect and the individual deviations (i.e., random) from that fixed effect. In general, the fixed effect describes normative and population-level developmental change over time, whereas the random effect describes individual differences in the starting point or change over time (however that manifests for a particular model). While all MEMs and SEMs are capable, in theory, of fitting both effects, there is often confusion about when design-based considerations might limit the ability to estimate complex effects at each level. Since three time points are needed to minimally identify a linear slope,^32^ we cannot typically^33^ estimate a random (i.e., individual) effect for anyone with fewer time points in our data (Parsons and McCormick, 2022). However, we might be able to fit a fixed effect in a linear (or even spline) model if we have more than two time points in our data in aggregate. Indeed, this is the entire rationale of accelerated longitudinal studies for covering large age ranges despite no single individual having more than a few observations and certainly no one observed over the entire age range in question (McCormick, 2021, McCormick et al., 2021, Sørensen et al., 2021a). Often in these designs, we can fix a relatively complex fixed effect of the developmental trajectory but be limited to a random intercept and/or linear slope (see here for an example).

##### 3.2.5.2 Generalizability

Relatively few of us are truly interested in describing the optimal developmental trajectory for the sample data we have at hand in a narrow way. Rather, we seek to use that data in a principled way to make inferences to a larger population. This desire for generalizability^34^ should serve as an important check on complexity when establishing the course of developmental change. It is almost axiomatically true that more flexible models, like GAMMs and latent-basis models, will provide better fit to any given sample, compared with more-restricted forms like the polynomial, given their sensitivity to local information (Wood et al., 2015). However, if we were to try to impose these same complex shapes on new sample data, it is likely that they would fail miserably, and re-estimating the effects would result in a new flexible shape. In contrast, a linear model might fit quite well across samples, even if it underperforms in each sample individually against alternatives. Model complexity should **always** be balanced against threats to external validity and generalizability because of this tendency to overfit. Researchers can take advantage of well-understood tools such as split-half and cross-validation (de Rooij and Weeda, 2020) in order to guard against this propensity for overfitting.

### 3.3 Covariates and distal outcomes

While in the previous section, we focused on establishing the course of development, we now turn to how different modeling frameworks accommodate understanding the causes (i.e., covariates/predictors) and consequences (i.e., distal outcomes) of developmental processes. We first address causes, detailing how different predictors enter the different models based on the level at which an effect operates. We pair this discussion with the idea of within- and between-person variance in longitudinal models, as well as an understanding of when a variable is properly understood solely as a cause versus a co-developing outcome. We then turn to the question of consequence by exploring how different modeling frameworks accommodate prediction based on trajectories. When we use the word “cause” here, we actually mean it without the usual soft-footing around with terminology. The structure and design of our data and the presence of unmeasured confounders determine how strongly these causal claims can be defended, but we do not think this means we should shy away from what single-headed arrows (i.e., regression rather than correlation) actually claim. The causal inference literature is vast on its own, but interested readers can see the following references for an entrée into the literature on longitudinal causal inference (Winship and Morgan, 1999, VanderWeele et al., 2016, van der Laan and Petersen, 2004).

#### 3.3.1 Covariates: Time invariant and time varying flavors

It should be clear^35^ that we do not treat the complexities of establishing the proper shape for developmental trajectories lightly. However, at a fundamental level, it is relatively unsatisfying to simply chart descriptively how development unfolds without developing causal explanations for those patterns.^36^ For instance, knowing that there is person-to-person variability in the rate of change in reward sensitivity begs the question: *why* might someone show more or less change in that sensitivity? These questions lead naturally to testable hypotheses about the predictive relationship between our outcome of interests and a variety of covariates that we can introduce into the model. These covariates come in two broad classes with surprisingly descriptive names (considering the usual trend in quantitative methods), *time-invariant* and *time-varying*. These different covariate classes enter the model at different levels and imply different types of causal processes. All of the modeling frameworks we consider here can broadly accommodate both types of covariates, although there are distinctions which we will highlight as appropriate. Furthermore, we will mostly discuss these considerations for a single covariate, but these principles naturally generalize to a set of predictors with little change.

Time-invariant covariates (TICs) are measures that do not vary across time (or at least the time window under consideration). The extent to which any measure is *truly* invariant is somewhat dubious, and so TICs are often variables that are measured once, and then strong assumptions (although often unrecognized) are made that they would not change if we were to measure them repeatedly.^37^ Other covariates are truly time-invariant (e.g., treatment group) or invariant-by-definition (e.g., childhood SES or maltreatment, maternal age at first birth). Regardless, TICs explain variance at the between-person level, which means that they explain person-to-person differences in the parameters of the growth model (e.g., intercept level or slope of change over time). In MEMs, this means that TICs enter the model equations at Level 2 (Curran and Bauer, 2011), or in SEMs that the TIC predicts the latent growth factor(s) directly (Biesanz et al., 2004). As such, their effect on the individual measures is transmitted through the random effects/latent factors^38^ (see here for code examples of each). In the SEMs, we can additionally predict specific repeated measures with a TIC (known as a multiple-indicator, multiple-cause or MIMIC model with direct effects; (Bauer, 2017, Jacobucci et al., 2019, Jöreskog and Goldberger, 1975, Kievit et al., 2014, Stoel et al., 2004), which sets up a form of mediation since the TIC now effects a repeated measure directly and indirectly (through the latent factor[s]). However, because there is no temporal precedence between the TIC and growth factors, this amounts to cross-sectional mediation in most cases (Curran et al., 2014, Curran and Bauer, 2011, Hamaker et al., 2014). SEMs also allow for the inclusion of latent TICs, where we can attenuate measurement error in the covariate as well (Bollen, 2002). Finally, we can include multiple TICs, as well as interaction terms, with reasonable ease (Curran et al., 2004, Curran and Bauer, 2011, Preacher et al., 2006). While this treatment may seem cursory, covariates at the time-invariant (or person) level are conceptually similar to standard regression contexts and their effects on the latent factors can be interpreted in much the same ways. For effects that incorporate time in more interesting ways, we need to turn to covariates which themselves show variability across time.

In a rare case of informative naming, time-varying covariates (TVCs) are covariates that...wait for it...vary over time. In this respect, they more closely resemble the repeated measures outcomes we are focused on when modeling developmental trajectories (more on this later) in our data frame, with multiple unique values for each individual. While TICs can only explain between-person variance, TVCs explain both within- and between-person differences depending on how they are entered into the model (Curran and Bauer, 2011). While perhaps unintuitive, we can think of TVCs as containing information unique to each time point (i.e., each individual measure) but also aggregate information (i.e., each person’s average over all measurements). To avoid making misattributions of effects at the wrong level, we need to take additional steps which we will discuss in the next section focused on separating variance. Like with TICs, we can include multiple predictors, as well as product terms. However, we can go further with TVCs by including a random component to the covariate effect, just as we do with the effects of time. The fixed effect of the TVC is the sample average effect, but the random component allows for individual differences in the relationship between the TVC and outcome. For instance, some individuals might show a stronger effect of anxiety on drinking than others. Furthermore, we might be able to bring TICs to bear to predict *which* individuals might show stronger or weaker effects of the TVC. This application of what are known as cross-level interaction effects (Bauer et al., 2006, Bauer and Curran, 2005, Curran et al., 2004) is relatively rare in the literature but offers a powerful tool for building causal explanations for the patterns of relationships we observe during development. There are well-developed tools in MEMs and SEMs for probing these and other forms of interaction that are very user-friendly (Preacher et al., 2006).

##### 3.3.1.1 Model comparisons

While the differences in how the various modeling frameworks treat TICs (which we mentioned above) are reasonably slight, there is a greater difference between how MEMs and SEMs treat TVCs. For MEMs, TVCs effects are aggregated across time to give a single effect estimate (unless some sort of formal interaction is included). This means that you can have an effect of anxiety that varies between individuals in magnitude (i.e., with a random effect), but you cannot probe time-specific effects of being higher or lower on a TVC at a *specific* time point. The closest you could come is to create a product interaction between time and the TVC to look at changes in the effect of the TVC across some smooth function of time. With SEMs, by contrast, we can get time-specific effects of the TVC and compare this with a model where those effects are held constant (i.e., the MEM form of the model; see here for an example).

Another difference arises when including lagged effects in MEMs versus SEMs. Lagged effects are often attractive because they ask how the prior level on a TVC prospectively predicts status on an outcome later in time. While not sufficient to establish causal effects (Rohrer and Murayama, 2021, Shadish et al., 2002), temporal precedence is a key condition in that pursuit. However, a lagged path creates implicit missing cases even if our data are otherwise complete, because there is no data on the prior level of the TVC before the first observation for each individual. Because of the way MEMs organize the data, this leads to listwise deletion of the first time point for each individual in the dataset, potentially causing significant issues with model estimation or power. For instance, many longitudinal datasets contain a maximum of 3 time points per person, so a lagged TVC MEM would render a random effect of time impossible at the individual level (McNeish and Matta, 2020). By contrast, the SEM is built from a system of equations, and it is trivial to just not include a path from this theoretically 0th observation of the TVC. Furthermore, SEM allows for a more flexible inclusion of individual information even with missing data on a covariate. Without digging too much into the technical details, MEMs and SEMs are fit by default with a conditional likelihood which does not allow for missing data on an exogenous (x-side) variable. However, with SEM software, we can implement a joint likelihood approach by estimating a mean and variance for the exogenous variable (Bauer, 2003, McNeish and Matta, 2020). This does invoke distributional assumptions that we otherwise do not make about exogenous variables but can be a way to preserve cases that have missing data on covariates.

##### 3.3.1.2 Decision tree III

**Fig. 3:**
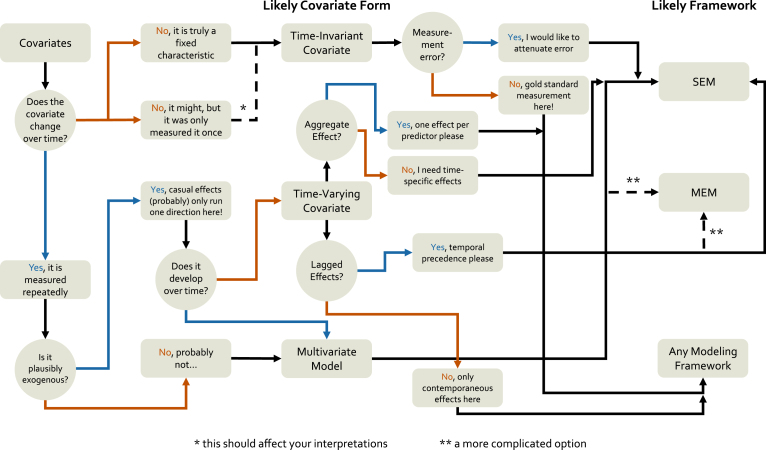
**A decision tree schematic for covariate modeling**. A heuristic for including covariate effects in longitudinal models. We not only focus on the type of covariate model one would likely implement but how they are handled in each of the modeling frameworks. Primary considerations include how the covariate changes – or does not – over time and whether we can plausibly consider it exogenous to the developmental system.

When adding covariates to our longitudinal models, we can consider three primary branching points (Fig. 3). First, whether the covariate obtains different values across time (time-varying) or is time-invariant (either in truth or by measurement limitations). Secondly, for TVCs, whether we need time-specific or lagged effects, where SEMs can provide a more tractable option compared to MLMs. Lastly, we need to consider whether we should treat our time-varying covariate as exogenous at all – either because it is systematically changing over time or because it shares reciprocal relationships with the primary outcome – versus including it as an additional outcome in a multivariate model.

##### 3.3.1.3 Separating within- and between-person variance

We mentioned previously that TVCs can explain within- and between-person variance because they contain time-specific and aggregate information. This represents a threat to internal validity since we might misattribute an effect as a within-person process (e.g., when I experience more stress, I take more risks) that is truly a between-person effect (e.g., individuals who experience more stress on average take more risks on average). Curran & Bauer (2011) have an excellent introduction to the issue and solutions in the MLM (which generalizes to MEMs), for those who wish a more in-depth treatment. Because most of our hypotheses in the behavioral and brain sciences concern within-person processes (Curran et al., 2014, Curran and Bauer, 2011, Hamaker et al., 2015), it is important to isolate those effects in our longitudinal models.

*MEMs*. Separation of within- and between-person variance in these models is accomplished through centering TVCs and the potential inclusion of person-level averages of TVCs (Curran and Bauer, 2011). While we will leave the details to the aforementioned treatment, the essential idea is that we can remove person-to-person variance in the TVC by subtracting the mean (which kind of mean will depend on the exact method; see Curran and Bauer (2011)) so that the TVC at Level 1 yields a pure within-person effect. We do not discard the average information, though, but instead create a new variable representing the person-to-person differences in average level of the TVC (which becomes a TIC) and enter it at level 2. Thus, we now estimate two different effects: (1) the pure within-person effect at Level 1, and (2) either the pure between-person effect at Level 2 (group-mean centering) or the difference between the within- and between-person effect (grand-mean centering). Interested readers can refer to prior work in this area (Curran and Bauer, 2011, McNeish and Matta, 2020) as well as a practical demonstration in the available code.

*SEMs*. Structural models can accomplish separation of within- and between-person variance using the same centering methods that we discussed with MEMs. The within-person effect is estimated with regression paths from the TVC directly to the repeated measures and between-person effect with paths to the growth factors. However, SEMs allow for another method of separation which nicely bridges to the next section. Rather than create new variables via centering, we can instead estimate a latent intercept factor on the TVC values just like we would with an intercept-only growth model (Hamaker et al., 2015), or add additional functional forms (e.g., linear slope; Curran et al., 2014). In this specification, all of the between-person variance is captured at the latent variable level, and all the within-person variance remains in the regression paths from the TVC to the outcome (see here for how to implement these models).

##### 3.3.1.4 Covariates versus multiple outcomes

Of course, once we have estimated a growth factor on the TVC, the natural question is: Is our TVC not a repeated measures outcome itself? The answer in a technical sense is “of course” since the factor predicts the TVC variables, but conceptually we might still think of the variable as an exogenous covariate rather than a fellow outcome. One operative question is whether we think that the covariate itself will change systematically with time. If it does, then failing to treat it as another outcome in a multivariate model will bias the effects of the TVC on the primary outcome of interest (Curran and Bauer, 2011, McCormick, 2021). However, an even more important, conceptual question, is whether we think our predictor is truly exogenous and the direction of causal effects only run in one direction, or whether the two (or more) constructs are co-developing across time (Curran and Hancock, 2021). We would suggest that most of the TVC effects we estimate in our science are the latter rather than the former.

The practical implementation of modeling a multivariate model is one of the sharpest dividing lines between MEMs and SEMs. MEMs are at their core, a univariate method; so while multivariate models are possible (Baldwin et al., 2014, Curran et al., 2023, MacCallum et al., 1997), it involves essentially tricking the model by combining the outcomes into a single variable and using dummy codes to separate the effects (see here). Furthermore, MEM software is not universally well-developed for modeling all the effects we would like in a multivariate model.^39^ SEMs, by contrast, are fundamentally a multivariate model (even in the single-variable case, it treats the repeated measures as separate outcome variables). As such, incorporating additional outcomes in the same model is trivial.^40^ In this sort of model, all sorts of effects are possible, including cross-construct regressions among the residuals (Curran et al., 2014, Curran and Hancock, 2021, Usami et al., 2019). Indeed, multivariate LCMs and LCSMs can be some of the most interesting models for testing developmental hypotheses (Curran and Hancock, 2021, Grimm et al., 2012, McArdle et al., 2009) and we would encourage researchers to consider them in their own work.

#### 3.3.2 Distal outcomes

Finally, we can turn to the final goal in the developmental sciences of characterizing the consequences of development. While our discussion sections are often full of the potential consequences of developmental trajectories, testing these hypotheses directly is still relatively rare. In some part, this is due to the challenges of collecting distal outcome data, since ideally this would temporally follow the data which is used to build developmental trajectories so that causal inferences are more sound. Not only is another wave of data collection an additional expense, but the temptation to also collect a full battery again rather than specific distal outcomes is not without merit. However, to fully contextualize development, we need to design studies that specifically test the consequences of individual differences in developmental trajectories. For instance, is variability in social information processing important for predicting later friendship, life-satisfaction or mental health outcomes? What about risky behavior or reward sensitivity during adolescence and contact with the criminal-justice system or physical well-being? If not, then it begs the question why we pour millions of dollars into our studies to map out behaviors that only ultimately impact adolescents’ lives through the CO_2_ our journals cost to host online.

While distal outcome estimation is an active area of research in quantitative methods (Smid et al., 2020, McCormick et al., 2023), when it comes to model selection, the differences between modeling frameworks are relatively clear. In general,^41^ MEMs need to utilize a two-step approach to estimate the distal outcome effects. This involves estimating the developmental effects and then using model-implied information in the form of Empirical Bayes estimates (Liu et al., 2021) in a second regression analysis with the distal outcome. This is not an ideal way to do distal outcome prediction because it treats model-implied information, which should have appropriate standard errors associated with it, as fixed-and-known (i.e., no uncertainty) in the regression analysis. It will be unsurprising to the reader at this point that the SEM methods can accomplish the prediction of the distal outcome with relative ease given the multivariate framework. The two step procedure is available through the estimation of factor score estimates, but has been shown to be sub-optimal (Skrondal and Laake, 2001) compared with simultaneous estimation of the entire model in most cases. As such, strong preference should be given to SEM methods in most models with distal outcomes in most cases (see here for examples of each approach).

### 3.4 Nested data

A final factor for model selection that we will consider here is what approaches exist to accommodate nesting in data. However, we will take a more expansive view of nesting than what is typically conceived and detail many ways in which we can incorporate grouping information into our longitudinal models. Broadly, we will take nesting to mean that some units in your data are grouped together into clusters which are more similar to one another than to members of other groups in the data. We will show that many different methods for incorporating this grouping information are possible, from simple, predictor-based adjustments, to building almost entirely separate models. Then, we will step back to consider when we need to adjust for nesting through formal model assumptions, like the standard MLM, versus alternatives.

#### 3.4.1 Methods for accounting for nesting

While often not considered a form of nesting, the inclusion of some categorical (e.g., binary, multinomial, or even ordinal) variable in a regression is a form of incorporating nested information into the model of our outcome of interest. Very common examples of this sort of approach include treatment effects, self-identified sex,^42^ or race/ethnicity variables into the model. Whether these are focal predictors or covariates used to partial out the associated variance, all of these methods account for conditional shifts in the mean of the outcome based on group membership. While there are likely exceptions to this general rule, these predictors are TICs and therefore explain person-to-person variability in the outcome, which is why we include them here as a method of accounting for nesting within our data. Furthermore, the multiple-groups model (Jöreskog, 1971) and its generalization to moderated nonlinear factor models (Bauer, 2017) can be viewed in the same light, but allow for any kind of parameter in the model to vary across either discrete (multiple groups) or continuous (MNLFA) variables (see the Nesting chapter for code examples).

More traditionally recognized forms of nesting (e.g., children nesting within schools, repeated measures nested within person, etc.) can broadly be accounted for using one of two general approaches: fixed and random effects. These methods account for the increased similarity of observations that are drawn from the same higher-level unit (e.g., school or the individual) compared to what we would expect in a simple random sample. This increased similarity actually reduces the amount of total information in our sample, since nested observations are partially redundant.^43^ The use of a fixed or random effect approach has historically been a matter of preference across disciplines (McNeish and Kelley, 2019, Hamaker and Muthén, 2020), however, we prefer to think of the two as complementary; to be used in conjunction depending on the specific needs of the model at hand. A fixed effect approach often^44^ involves the inclusion of dummy code predictors for each group directly into the model equation (McNeish and Kelley, 2019). If we had 3 groups in our data (perhaps 2 treatment groups and a control), the model expression would look something like the following:

(12) $$y_{ti}=\beta_{1}\mathrm{Cntrl}+\beta_{2}{\mathrm{Treat}}_{1}+\beta_{3}{\mathrm{Treat}}_{2}+r_{ti}$$

Here we drop the traditional intercept ($\beta_{0}$) and model the effect of each group using an absolute coding scheme (we could alternatively drop $\beta_{1}$ and use a reference scheme; McNeish and Kelley, 2019). This has the powerful effect of removing group differences in the conditional mean of $y_{ti}$ based on group (which is exactly what we do with our group predictors in the first example and why we include it). However, the fixed-effect approach can take this idea even further by removing all group differences in the effect of other predictors of interest by use of interactions. So, if we were to include time as a predictor now, and wanted to assess the effects of each group, the model expression would take the following form (see code examples for implementation).

(13) $$y_{ti}=\beta_{1}\mathrm{Cntrl}+\beta_{2}\left(\mathrm{Cntrl}\times \mathrm{Time}\right)+\beta_{3}{\mathrm{Treat}}_{1}+\beta_{4}\left({\mathrm{Treat}}_{1}\times \mathrm{Time}\right)+$$

$$\beta_{5}{\mathrm{Treat}}_{2}+\beta_{6}\left({\mathrm{Treat}}_{2}\times \mathrm{Time}\right)+r_{ti}$$

Here we must include a new product term for each group in order to model the effect of time within that group. You can see how this fixed-effect approach can easily get quite verbose with the addition of new predictors or in cases with many more groups. As such, this approach may not be ideal for cases where we wish to model many groups or where groups are small (e.g., kids nested within families being a good example of both issues). However, the fixed-effect approach is likely best suited for situations where the higher-level unit is more a practical feature of data collection rather than of particular theoretical interest. Canonical examples of this might be large, multi-site studies where data collection occurs in proximity to participating universities (e.g., ABCD) and school-based assessments in a local community, or where we have an exhaustive countable list of groups like countries or religious groups. In the former cases, we are less interested in generalizing our findings specifically to some population of assessment sites per se (we want to generalize to the population of people, not sites), but we do want to control for site-to-site differences in a whole host of factors (e.g., recruitment/implementation strategies, scanner features, etc.). In the latter, we have the full population of groups and we can make valid inference to them directly. Under these circumstances, the fixed-effects approach is well-suited because it removes all sources of variance due to group differences without requiring us to know each of the relevant factors that cause the differences between groups, and inferences are restricted to the groups we observe directly rather than generalizing to a larger population (McNeish and Kelley, 2019).

The random-effect approach, exemplified in the MLM,^45^ takes a different approach to nested data, which allows for some desirable inferential advantages at the price of additional assumptions. A key assumption is that groups we observe in our data are random draws from some larger population of groups we *might* have observed if we were to perform the study repeatedly (McNeish et al., 2017). Nesting within families or individuals (for longitudinal data) are good examples of groups that might fit this assumption; we are unlikely to get the exact same groups if we were to re-sample (in contrast to something like assessment sites or religious groups where we *would* expect to draw the same groups again). Another assumption we make with random effects is that the unit-specific effects are normally distributed in the population. This typically requires a larger number of groups than we would typically use with a fixed-effect approach, although random-effects models can be fit with smaller numbers of clusters if appropriate care is taken (McNeish and Stapleton, 2016). While random-effect models are broadly popular in the behavioral and brain sciences, some have argued that the additional assumptions, which when violated lead to biased effects, are not warranted in many applications and advocate for other, distribution-free approaches (McNeish et al., 2017).

#### 3.4.2 Nesting versus cluster correction

When higher-level nesting is present in longitudinal data (e.g., repeated measures within kid within family), it is a natural inclination to default to the MLM (or MEMs more generally). More recently, retaining the SEM framework has become more popular through the multilevel-SEM (MLSEM) approach (Muthén, 1989, Preacher et al., 2010), although the cluster-level sample size requirements are large Hox and Maas, 2001. However, alternatives do exist for correcting, rather than modeling, higher levels of nesting that may be of interest. For instance, cluster-corrected standard errors account for the dependence in the data when performing inferential tests (see here for examples). This correction approach may be a viable alternative to formal nesting under reasonably common conditions where we have higher levels of nesting and do not wish to ignore it, but we do not have substantive hypotheses about causal relationships at that higher level McNeish et al., 2017. In our example, we would almost certainly wish to account for within-family similarity when modeling adolescent trajectories of risky behavior. However, we might have no hypotheses about predictors that influence family-level factors. In this case, the nested structure at the family level is more of a nuisance we are trying to control for, and might not be worth the additional assumptions of modeling a random intercept of family McNeish et al., 2017, McNeish and Wentzel, 2017. The correction approach may be especially useful for situations where the random effect structure is already quite complex and higher-level variance components are likely to be relatively small — and therefore challenging to estimate.

## 4 Conclusions

And another thing...no, we promise this is the end. The choice of modeling approach for longitudinal data is a complex one; any one of the sections we outlined here could (and indeed are) be the subject of their own specialized primer. From the coding of time to dealing with clustering among observations, we have seen the various strengths and limitations of the four modeling frameworks and hopefully provided guidance for researchers wishing to apply these models in their own substantive research.

### 4.1 Model fitting versus model planning

In much of the primer, we discussed different modeling options with the implicit assumption that the primary audience for this primer is someone who has data and wants to know what to do with it. However, we would highlight the role that all of the considerations and comparisons we explored here can and should play in informing future longitudinal data collection. By their nature, longitudinal studies simply take a lot of *time*; therefore, having a well-reasoned idea of which modeling framework will best test the theoretical question of interest should affect how data are collected — ideally to maximize the power of the model to give you a meaningful answer. For instance, if we were testing a theory that suggests that two variables co-develop over time, we would likely want to choose a more consistent assessment schedule to maximize our ability to use SEM models that more easily handle multivariate outcomes. By contrast, if we needed to test a theory which posits a highly-nonlinear developmental trajectory, we likely want to use an accelerated design to achieve enough age heterogeneity to fit a complex piecewise or GAMM model. Having a concrete idea about the modeling options available *before* data collection allows us to match models to theory rather than having to accommodate suboptimal data structures at the modeling stage.

### 4.2 Revisiting aims

Overall, we aimed to provide researchers with a heuristic system of guideposts (Aim 1) for selecting among competing models to take advantage of the advanced longitudinal modeling approaches developed across many disciplines to best test their developmental theory. By necessity, we have likely smoothed over additional complexity and left out yet more considerations that could be raised in model selection for repeated measures data, however, we also provide extensive reference to prior work (Aim 2), with a focus on both the foundational quantitative methodological development work and practical examples of longitudinal modeling in developmental neuroimaging data (and an associated codebook companion; Aim 3). With these tools, we hope to not only equip researchers with the tools and knowledge necessary to apply longitudinal models but also to shape decisions for subsequent longitudinal data collection with specific models in mind that will power future discoveries concerning the mechanisms of change across development.

## Appendix A Supplementary data

MMC S1 The supplementary material provides a guide for reading equations, a list of common abbreviations, and a symbol guide.

## Footnotes

1 MLMs allow for more complex types of nesting, however, we focus on those common in longitudinal models here.

2 For now, we will use “time” as a stand-in for any developmental process that we might use as a predictor in a longitudinal model. This could be something intuitive and simple like age or wave of assessment, or more abstract — such as maturation as indexed through pubertal status. Especially in MEMs, we have a lot of flexibility about what “time” is. See our discussion here (Section 3.1.4.1) for more details.

3 Note that while the model does not **require** us to have a random slope, it is relatively uncommon to only have a fixed slope in longitudinal models.

4 This might sound like a residual, which is exactly what it is. Typically, we reserve “residual” for the level 1 deviation, and “random effects” for the level 2 deviations.

5 A delightfully vague standard as “sufficient” is impacted by many considerations (e.g., model complexity, higher order nesting, etc.).

6 Who says math cannot be whimsical?

7 In SEM terminology, items are often called “indicators” of the latent variable, but we will retain “item” here for readers who may be less familiar with these conventions.

8 We need to distinguish between the intercept **of** a factor (an unconditional or conditional mean depending on the model, denoted by  ) and the factor that represents the intercept of the trajectory (typically denoted by  ).

9 Yes, we have in fact introduced another term for a residual. Welcome.

10 Lowercase theta (θ) represents the vector of model parameters while uppercase theta (Θ) represents the residual covariance matrix (blame the Greek alphabet for not having more characters).

11 This is an **incredibly** over-simplified treatment of model fit, the relevant citations go into much greater detail.

12 These definitions may seem trivial, but bear with us; this enables us to do some cool things with the LCSM.

13 We emphasize this to highlight that they are not “similar” or “close enough” but literally indistinguishable.

14 Here we put residual in quotes because it has all the properties of the original variable which is uncommon for a residual in other contexts.

15 The degree to which this reflects the reality of the observation schedule is a function of recruitment and scheduling.

16 Of course, the reader can likely already see an alternative way to parameterize time in such a study, but we will return to this in a moment.

17 Planned missingness is a form of Missing Completely at Random (MCAR) which is the super-duper special form of the Missing at Random (MAR) assumption needed for unbiased model estimation. This of course does not preclude other, more pernicious, forms of missingness that **will** bias model estimation.

18 As well as for the covariance between the intercept and slope.

19 Of course, this depends on random variability in the slope estimates since simpler models might give different fixed but not random effects (random intercept, fixed slope) or identical fixed and random effects (random intercept-only).

20 Mixed-effects models can still suffer when data coverage over certain age ranges is limited. One person out at 80 years old (young at heart?) in a study of adolescents will not allow you to make appropriate lifespan inferences.

21 There are also continuous time SEM approaches that we will not address here but may be of interest for dealing with fully inconsistent assessment schedules (e.g., Oud and Jansen (2000-06-01)).

22 Remember that mixed effects models really are just specialized latent variable models so this convergence should not be surprising.

23 In principle, some sort of parameter moderation (Bauer, 2017) by individually-varying assessment at the level of the latent time-specific or change factors could be possible but we have not encountered such a model in the wild.

24 In other words, one of the authors (we will let you guess which) is somewhat long-winded.

25 More exotic residual structures – e.g., autoregressive or Toeplitz – that are often included in intensive longitudinal models are uncommon in traditional longitudinal models where enough time passes between observations that residual dependence decays towards zero. Because of that, we will only dwell on diagonal residual options – where there are no correlations between residuals of different items.

26 The notable exception being *lmer* from the *lme4* R package, which does not allow for complex residual structures. To obtain access to the heteroscedastic residual specification, use *lme* from the *nlme* package.

27 Although it would be somewhat of a waste of a GAMM’s utility, you can easily specify a linear effect of time with no spline.

28 Linear being time^1^, quadratic being time^2^, cubic being time^3^, inverse being time^−1^, and so forth.

29 Right? Right...????

30 It can be understandably maddening for those new to quantitative methods that terms like nonlinear model can refer to multiple things. Here we will mostly use it to refer to nonlinearities in the pattern of change over time being modeled as opposed to models where the parameters enter the equation nonlinearly (e.g., models for categorical outcomes). However, in the interest of being maximally confusing, there are methods which are nonlinear in both senses (e.g., Gompertz curves).

31 Here we mean that the researcher is interested in predicting an outcome without offering a specific causal explanation of how or why an effect is predictive. These models are very common in machine learning applications but less so in the psychological or brain sciences.

32 And 4 to specify a quadratic, etc.

33 We can sort of approximate a random slope effect with 2 time points per person, but it is really more of a random difference score which will be less reliable since the change is determined between 2 points instead of estimated like with 3+.

34 The arguably more important, if oft-neglected, sister of reproducibility and replicability. There is a band name in there somewhere.

35 If nothing else than by the amount of (virtual) ink we spilled on the topic in the prior section.

36 While covariates might also be useful for *purely* predictive modeling where we are unconcerned with explanations and only minimizing the prediction error, another branch of models entirely are useful for those sorts of aims and so we will not spend time on those applications. It should also be noted that those models are not exempt from causal and explanatory concerns, but they manifest differently – e.g., in which variables are selected for prediction.

37 An oft-discussed example of this is the inclusion of sex/gender variables as TICs. We do not want to gloss over the challenge; gender is clearly not immutable across time, but it is possible that within a sample there is not sufficient variability to model time-varying effects. We think that these are serious questions that should inform study design (e.g., sampling, using time-varying measures of gender expression instead of categorical measures) as those will determine the possibilities for modeling.

38 Remember that these are really the same thing (Curran, 2003, Bauer, 2003).

39 As of this writing, the popular R packages do not allow for unique time-specific residual covariances, which are an important feature of multivariate models. To our knowledge, only SAS PROC MIXED allows for complete flexibility in modeling all the effects we could get with ease in any SEM software. Note that for these purposes, we consider Mplus to be a SEM software because its MEMs are implemented in a SEM convention.

40 You have got to love it when a quantitative person says this. But it should be!.

41 Fully Bayesian methods complicate this distinction somewhat, but as with the Mplus implementation of MLMs, this is really more of a full latent variable framework and is similar to SEM estimation approaches in that respect.

42 To echo an earlier footnote, this practice is likely not ideal for capturing the full range of sex and/or gender effects, but the constraints of current datasets mean that it is often done in practice.

43 The degree of redundancy is determined by the intra-class correlation of observations within a unit.

44 Alternative approaches might involve cluster-mean centering predictors (see Hamaker and Muthén (2020) for a more detailed exposition of these methods).

45 While not as easily apparent, the LCM accommodates the nesting of observations within individuals in an equivalent way to the MLM growth model (Curran, 2003).
