# Supplementary material for: The Hitchhiker’s guide to longitudinal models: A primer on model selection for repeated-measures methods
Source: Dev Cogn Neurosci. 2023 Jul 26;63:101281. doi: 10.1016/j.dcn.2023.101281 (PMC10412784; doi:10.1016/j.dcn.2023.101281)
Supplement: MMC S1 — The supplementary material provides a guide for reading equations, a list of common abbreviations, and a symbol guide. [file mmc1.pdf]

1 Matrix Conventions

Matrix expressions are a powerful and compact way to arrange information with great computational advantages. However, many researchers are familiar with simple equations for only a single outcome that can be written on a single line – we will call these scalar expressions – and matrix expressions are often foreign. Here we attempt to clarify the notational conventions for matrix expressions. We will highlight these conventions using a set of simple ordinary least squares (OLS) regression equations.

The most familiar form of an equation is in *scalar* form, where each term either refers to a single estimated parameter, or to a single observed variable in our data frame. Scalar here just refers to individual numerical values. For instance, in a single-predictor regression, we have the following:

$$\underbrace{y_i}_{\text{observed variables}} = \overbrace{\beta_0 + \beta_1}^{\text{estimated parameters}} \underbrace{x_i}_{\text{observed variables}} + \overbrace{\varepsilon_i}^{\text{residual}} \tag{1}$$

Where i indicates that each individual has a different value of y and x. Note that all the terms in this equation are lower-case and italicized. This is the general notation rule for all scalar expressions. A matrix equation instead represents a set of scalar equations for multiple outcomes, which are arranged in such a way that matrix multiplication gives us all the scalar expressions we want. When we move to a matrix, we replace the italicized lower-case scalar expressions with two different kinds of notation. The first is a **vector** – a single-dimension arrangement of variables or parameters – which we denote with a lower-case, bold-face letter or symbol. For instance, in Equation 1, we have a single scalar predictor, x_i. In a multiple regression, however, we have many scalar predictors, each with their own regression coefficient, that would look something like this:

$$y_i = \beta_0 + \beta_1 x_{1i} + \beta_2 x_{2i} + \beta_3 x_{3i} + \varepsilon_i \tag{2}$$

Instead of writing each regression coefficient out in this scalar expression, we could collect them into a vector like so:

$$\overbrace{\boldsymbol{\beta}}^{\textbf{vector}\text{ of }\beta\text{ coefficients}} = \begin{bmatrix} \overbrace{\beta_0}^{\text{individual } \textit{scalar} \text{ coefficient}} \\ \beta_1 \\ \beta_2 \\ \beta_3 \end{bmatrix} \tag{3}$$

Positions in a vector are indicated by row, so $\boldsymbol{\beta}[2]$ indicates β_1 and so forth. The other notation is the **MATRIX** – a multiple-dimension arrangement of variables or parameters – which we denote with an upper-case, bold-face letter or symbol. For instance, the set of predictors (x's) in Equation 2 can be arranged into different columns for each predictor and different rows for each individual $(1 - n)$. This would take the following form:

scalar value of x_3 for person $(i = 1)$

MATRIX of predictors

$$\mathbf{X} = \begin{bmatrix} x_{1,1} & x_{1,2} & x_{1,3} \\ x_{2,1} & x_{2,2} & x_{2,3} \\ \vdots & \vdots & \vdots \\ x_{n,1} & x_{n,2} & x_{n,3} \end{bmatrix} \tag{4}$$

column **vector** of x_{1i}

All values within this matrix can be indicated by a combination of their row position (first dimension) and column position (second dimension), with the notation $\mathbf{X}[row, column]$. For instance, $\mathbf{X}[1,3]$ indicates the value of the first person on the third predictor (indicated in Equation 4).

While this is not an exhaustive review of matrix algebra, this should help to guide the reader through notational type-face of many of the equations in the main text. For a fuller explanation of matrix algebra and how to express multiple regression in matrix terms, many online resources exist (e.g., here).

We can demonstrate this in a slightly more complex example that we might encounter in longitudinal models. Below we can write out the matrix equation for a conditional linear latent curve model (LCM) with 4 time points. This means that we will have two latent growth factors which are regressed on an exogenous covariate. The general matrix equation for this model would resemble the following (see tables in Section 3 for more explanations on what each term means):

$$\begin{aligned} \mathbf{y}_i &= \boldsymbol{\Lambda}\boldsymbol{\eta}_i + \boldsymbol{\varepsilon}_i \\ \boldsymbol{\eta}_i &= \boldsymbol{\alpha} + \boldsymbol{\Gamma}\mathbf{x}_i + \boldsymbol{\zeta}_i \end{aligned} \tag{5}$$

We can go through and recognize which terms are **vectors** and **MATRICES** based on the formatting conventions we have outlined previously.

vector of observed outcomes; **vector** of latent factors

$$\mathbf{y}_i = \boldsymbol{\Lambda} \; \boldsymbol{\eta}_i + \boldsymbol{\varepsilon}_i$$

MATRIX of factor loadings; **vector** of residuals

(6)

vector of factor means; **vector** of observed covariates

$$\boldsymbol{\eta}_i = \boldsymbol{\alpha} + \boldsymbol{\Gamma} \; \mathbf{x}_i + \boldsymbol{\zeta}_i$$

MATRIX of regression coefficients of $\boldsymbol{\eta}_i$ on $\mathbf{x}_i$; **vector** of disturbances

We can finally expand each of these vectors and matrices to show how each *scalar* value would be arranged, resulting in the following:

$$\begin{bmatrix} y_{1i} \\ y_{2i} \\ y_{3i} \\ y_{4i} \end{bmatrix} = \begin{bmatrix} 1 & 0 \\ 1 & 1 \\ 1 & 2 \\ 1 & 3 \end{bmatrix} \begin{bmatrix} \eta_{1i} \\ \eta_{2i} \end{bmatrix} + \begin{bmatrix} \varepsilon_{1i} \\ \varepsilon_{2i} \\ \varepsilon_{3i} \\ \varepsilon_{4i} \end{bmatrix}$$

$$\begin{bmatrix} \eta_{1i} \\ \eta_{2i} \end{bmatrix} = \begin{bmatrix} \alpha_1 \\ \alpha_2 \end{bmatrix} + \begin{bmatrix} \gamma_1 \\ \gamma_2 \end{bmatrix} \begin{bmatrix} x_{1i} \end{bmatrix} + \begin{bmatrix} \zeta_{1i} \\ \zeta_{2i} \end{bmatrix} \tag{7}$$

2 Common Abbreviations (in general order of appearance)

MEM Mixed-Effect Model

MLM Multilevel Model

GAMM Generalized Additive Mixed Model

OLS Ordinary Least Squares

AIC Akaike Information Criterion

BIC Bayesian Information Criterion

SEM Structural Equation Modeling

LCM Latent Curve Model

LCSM Latent Change Score Model

ARCL Autoregressive Cross-Lag Panel Model

GMM Growth Mixture Models

CFI Comparative Fit Index

TLI Tucker-Lewis Index

RMSEA Root Mean Square Error of Approximation

CFA Confirmatory Factor Analysis

FIML Full Information Maximum Likelihood

TIC Time-invariant Covariate

TVC Time-varying Covariate

MNLFA Moderated Nonlinear Factor Analysis

MLSEM Multilevel Structural Equation Modeling

3 Common Symbols/Notations and their Definitions

3.1 Mixed-Effects Models (MEM) Terminology & Notation

Many things are common across both multilevel and generalized additive mixed models. Hence, we will cover most notations under the banner of the MLM (because that is how it is encountered in the manuscript) and then note the unique additional notations for the GAMM.

3.1.1 Commonly-Encountered Terms

Level 1 Model equations that structure the within-unit data and effects. In longitudinal models, this level typically models the repeated measures within individuals (i.e., time-varying).

Level 2 Model equations that structure the between-unit data and effects by writing additional equations for Level 1 parameters. In longitudinal models, this level typically models the average differences between individuals (i.e., time-invariant).

Reduced Form Equation This model expression is formed by substituting Level 2 equations into the Level 1 equations. While Level 1 and 2 are useful conceptual tools, this is how the model is actually implemented and estimated in software.

Fixed Effects Average effects across clusters (typically individuals in longitudinal models).

Random Effects Cluster (individual)-specific deviations from the fixed effects. Random effects are assumed to follow a normal distribution.

Homoscedastic Residuals Used to refer to a model where the residual variances of the repeated measures outcome are constrained to be equal across time. This is the typical default for mixed-effects models.

Heteroscedastic Residuals Used to refer to a model where the residual variances of the repeated measures outcome are estimated uniquely for each measurement occasion.

Spline Nonlinear smoother that are used to model nonlinear relationships between a predictor and outcome. These functions are constructed by combining multiple simpler functions, such as polynomial or trigonometric functions, in a way that allows them to be flexibly adapted to fit the data.

Knot Point A transition point between two simple functional forms when building the overall functional form.

"Wiggliness" A measure of the squared change in instantaneous slope across the spline function. Essentially the number of bends that characterized the unknown functional form.

Overfitting When a model is overly complex and has too many parameters relative to the amount of training data available. This can lead the model to learn the noise or random fluctuations in the training data, rather than the underlying pattern or relationship that the model is intended to capture. As a result, the model may perform well on the training data, but it will not generalize well to new data and may make inaccurate predictions or classification decisions.

3.1.2 Multilevel Models (MLM)

t	Level 1 unit identifier (in longitudinal models, most often the measurement occasion or age, but can also be many other things), $t = 1, 2, \ldots, T$.
i	Level 2 unit identifier (in longitudinal models, most often the individual), $i = 1, 2, \ldots, N$.
N	Total sample size of Level 2 units (in longitudinal models, typically the number of individuals).
y_{ti}	Observed outcome for person i at time t.
x_{ti}	Level 1 observed predictor for person i at time t.
β_{pi}	Regression coefficient (beta) in Level 1 equation for the pth predictor at Level 1. These coefficients begin at 0 for the intercept (e.g., $y_{ti} = \beta_{0i} + \beta_{1i}x_{ti} + r_{ti}$) where the coefficient may or may not vary over individuals (or other clusterings) as indicated in the Level 2 equation by the in- or exclusion of a random effect.
r_{ti}	Level 1 residual for person i at time t (unexplained variability at Level 1). These are analogous to the item residuals (ε) in the SEM growth model.
σ^2	Level 1 residual variance estimate (sigma; i.e., the variance of r_{ti}). These are analogous to the item residuals (θ) in the SEM growth model.
γ_{pi}	A **fixed effect** (gamma; constant magnitude across all t and i) indicating the average effect (in a linear model) of a Level 1 β coefficient across Level 2 units. These are analogous to the factor means (α) in the SEM growth model.
u_{pi}	A **random effect** (unexplained variability at Level 2) indicating deviations from γ across Level 2 units. These are analogous to the factor disturbances (ζ) in the SEM growth model.
τ	A variance or covariance parameter (tau) for the random effects (e.g., τ_{00} is the variance of u_{0i}, τ_{11} is the variance of u_{1i}, and τ_{10} is the covariance between u_{0i} and u_{1i}). These are analogous to the ψ parameters in the SEM growth model.
$\mathbf{T}$	The covariance matrix for random effects, e.g., $\mathbf{T} = \begin{bmatrix} \tau_{00} & \\ \tau_{10} & \tau_{11} \end{bmatrix}$. Analogous to the $\mathbf{\Psi}$ matrix in the SEM growth model.
ICC	Intraclass correlation, a measure of dependency in the data, indicating the extent to which Level 1 observations within a Level 2 unit (i.e., here repeated observations within a given individual) are correlated. Also interpretable as the proportion of variance in $y_t i$ that is attributable to between-cluster differences in mean levels of the outcome. Obtained from a random-effects ANOVA model (an MLM with no predictors and a random intercept) as $\frac{\tau_{00}}{\tau_{00}+\sigma^2}$.

3.1.3 Generalized Additive Mixed Models (GAMM)

$f()$	A stand-in expression that indicates that the relationship between x and y can have any unknown functional form. Often this overall unknown functional form is built up from a set of simpler, known functional forms (e.g., splines).

3.2 Structrual Equation Models (SEM) Terminology & Notation

Like with the MEM notation, many things are common across both latent curve and latent change score models. Hence, we will cover most notations under the banner of the LCM (because that is how it is encountered in the manuscript) and then note the unquie additional notations for the LCSM.

3.2.1 Commonly-Encountered Terms

Endogenous Variables with causes specified "inside" the model; or those which appear on the left-hand side of at least one equation. Alternatively referred to as outcome or dependent variables.

Exogenous Variables with causes specified "outside" the model; or those which only appear on the right-hand side of equations. Alternatively referred to as predictors, covariates, or independent variables.

Random Effects Cluster (individual)-specific deviations from the fixed effects. Random effects are assumed to follow a normal distribution. This is the typical default for structural equation models.

Homoscedastic Residuals Used to refer to a model where the residual variances of the repeated measures outcome are constrained to be equal across time.

Proportional Change Predicting latent change from prior status (often denoted as β).

3.2.2 Latent Curve Model (LCM)

N	Total sample size.
i	An individual sample unit, where $i = 1, 2, 3, \ldots, N$
L	Likelihood of the data given the specified model.
ℓ	Log-likelihood, or the natural log (ln) of the likelihood.
T	Test statistic comparing a specified model to a baseline comparison model.
ν	An intercept (nu) of an observed outcome. These are analogous to the β_0 intercept mixed-effect growth model (or any standard regression equation).
$\boldsymbol{\nu}$	A vector of item intercepts, where the length of the vector is equal to the number of observed items.
λ	A factor loading (lambda). These typically* represent the regression of an observed outcome on a latent factor. They are interpreted just like regression coefficients: "A 1-unit change in the latent factor is associated with a λ-unit change in the outcome."
$\boldsymbol{\Lambda}$	A matrix of factor loadings, where the number of rows is equal to the number of observed items, and the number of columns is equal to the number of latent factors.
η	A latent factor/variable (eta). These are variables for which we do not have observed values in our data, but rather we infer their existence from the pattern of covariance between observed items.

*You can have factor loadings between two latent variables in a "higher-order" factor model.

$\boldsymbol{\eta}$	A vector of latent factors, where the length of the vector is equal to the number of latent factors.
ε	A residual for an observed item (epsilon). These are analogous to r_{ti} in the mixed-effects growth model.
$\boldsymbol{\varepsilon}$	A vector of item residuals, where the length of the vector is equal to the number of observed items.
θ	The variance or covariance (theta) of the residual ε. We need to be careful, because theta gets used multiple times, but here we use the italicized, normal-face version. These are analogous to σ^2 in the mixed-effects growth model.
$\boldsymbol{\Theta}$	The covariance matrix of item residuals. In most traditional growth models, this matrix is typically diagonal, which means that we only estimate residual variances (on the diagonal – hence the name) and no residual covariances, e.g., $\boldsymbol{\Theta} = \begin{bmatrix} \theta_{11} & \\ 0 & \theta_{22} \end{bmatrix}$
α	An intercept (alpha) of a latent factor. These can either represent the mean of the latent factor, or the intercept (again think β_0) if the factor is the outcome of another variable. These are analogous to the fixed effects (γ) in the mixed-effects growth model.
$\boldsymbol{\alpha}$	A vector of factor means or intercepts, where the length of the vector is equal to the number of latent factors.
ζ	A disturbance (or residual) of a latent factor (zeta). These are analogous to the random effects (u) in the mixed-effects growth model.
$\boldsymbol{\zeta}$	A vector of factor disturbances, where the length of the vector is equal to the number of latent factors.
ψ	The variance or covariance (psi) of the disturbance ζ (e.g., ψ_{11} is the variance of η_{1i}, ψ_{22} is the variance of η_{2i}, and ψ_{21} is the covariance between η_{1i} and η_{2i}). These are analogous to the τ parameters in the mixed-effects growth model.
$\boldsymbol{\Psi}$	The covariance matrix of latent factor disturbances. e.g., $\boldsymbol{\Psi} = \begin{bmatrix} \psi_{11} & \\ \psi_{21} & \psi_{22} \end{bmatrix}$. Analogous to the $\mathbf{T}$ matrix in the multilevel growth model.
γ	A regression coefficient of a latent factor or observed variable at the structural level on an exogenous covariate (gamma).
$\boldsymbol{\Gamma}$	A matrix of regression coefficients where the number of rows is the number of latent factors or observed variables at the structural level and the number of columns is the number of exogenous covariates.
β	A regression coefficient between two variables (either observed or latent) at the structural level of the model (beta).
$\mathbf{B}$	A matrix of regression coefficients where the number of rows and columns is equal to the number of endogenous variables at the structural level of the model.
$\boldsymbol{\theta}$	The vector of all model parameters (i.e., means/intercepts, variances, and covariances).

$\hat{\boldsymbol{\theta}}$	The vector of the model-implied estimated values of all the parameters in $\boldsymbol{\theta}$
$\mu(\hat{\boldsymbol{\theta}})$	A vector of model-implied means of the observed variables in the model.
$\Sigma(\hat{\boldsymbol{\theta}})$	A matrix of the model-implied variances and covariances of the observed variables in the model.

3.2.3 Latent Change Score Model (LCSM)

$\Delta\eta_{t,t-1}$	Latent difference factor, typically denoted with the subscript of later (t) then earlier ($t-1$; it may or may not include the comma), e.g., $\Delta\eta_{21}$ is the latent difference between observation 2 and 1.
β	The regression coefficient between the latent difference score and the prior time point variable (e.g., $\Delta\eta_{21}$ on y_1). While it is ultimately a regression coefficient (like β_1 in a standard regression equation), the LCSM often uses this as a special notation for this specific parameter.

3.3 Time Structure

Cohort Study A highly consistent form of assessment schedule where individuals are assessed repeatedly at (roughly) the same ages and intervals.

Cohort-Sequential Study A hybrid assessment schedule where individuals within a given cohort are assessed repeatedly at (roughly) the same ages and intervals, but where separate cohorts begin assessments at different ages. Also known as multi-cohort studies.

Accelerated Study A highly inconsistent form of assessment schedule where individuals are initially assessed across a continuous range of ages and then followed up at regular or irregular intervals.

3.4 The Shape of Development

Polynomial Function A functional form composed of terms raised to a power (e.g., x^1,x^2, etc.). In the context of longitudinal models, these are easily specified functional forms where each of the values of the polynomial predictor is fixed and known.

Piece-Wise Function A functional form composed of one or more polynomial splines joined together at a knot point.

Nonlinear Function A functional form which does not follow a straight line over time. This class of functional forms is highly varied, including higher-order polynomials (although we distingush those from other nonlinear forms for reasons explained elsewhere), exponential, sinusoidal, and exponential forms. A model can also be nonlinear "with respect to the parameters", where parameters are combined in ways other than simple addition and includes models for discrete outcomes, as well as many models for nonlinear functional forms (e.g., sigmoidal curves).

3.5 Covariates and Distal Outcomes

Time-Invariant Covariate A covariate with a fixed value over time (at least within the range of observation). These covariates enter the model at the between-person level (or Level 2) and explain average between-person differences (or between-cluster differences more generally).

Time-Varying Covariate A covariate with values which can change over time. These covariates enter the model at the within-person level (or Level 1) and explain occasion-to-occasion differences (or within-cluster differences more generally).

Distal Outcome An outcome which (ideally) exists at some temporal distance to the growth process being model and is predicted from individual differences in the growth process. Often used to assess the consequences of these differences for long-term outcomes.

3.6 Nested Data

Multiple-Groups Model A model where some subset of model parameters are allowed to vary across discrete groups.

Moderated Nonlinear Factor Analysis A model where some subset of model parameters are allowed to vary across either discrete groups or continous covariates. The multiple-groups models is a special case of this model.
